# Bitter fruits of hard labour: diet metabarcoding and telemetry reveal that urban songbirds travel further for lower-quality food

**DOI:** 10.1007/s00442-020-04678-w

**Published:** 2020-06-13

**Authors:** Crinan Jarrett, Luke L. Powell, Heather McDevitt, Barbara Helm, Andreanna J. Welch

**Affiliations:** 1 Institute of Biodiversity, Animal Health and Comparative Medicine, University of Glasgow, Glasgow, UK; 2 Department of Biosciences, Durham University, South Road, Durham, UK; 3 GELIFES-Groningen Institute for Evolutionary Life Sciences, University of Groningen, Groningen, The Netherlands

**Keywords:** Urbanisation, Provisioning, Reproduction, Blue tit, Faecal

## Abstract

Rapidly increasing urbanisation requires mitigation against associated losses of biodiversity and species abundance. In urban-breeding birds, altered food availability for nestlings is thought to reduce reproductive success compared to forest populations. To compensate for shortages of preferred foods, urban parents could increase their search effort for optimal diets or provision other foods. Here, we used telemetry and faecal metabarcoding on blue tits from one urban and one forest populations to compare parental effort and comprehensively describe nestling diet. Urban parents travelled on average 30% further than those in the forest, likely to offset limited availability of high-quality nestling food (i.e. caterpillars) in cities. Metabarcoding, based on a mean number of 30 identified taxa per faeces, revealed that the diets of urban chicks were nonetheless substantially shifted to include alternative foods. While in the forest caterpillars comprised 82 ± 11% of taxa provisioned to nestlings, in the city they constituted just 44 ± 10%. Pre-fledging chick mass as well as offspring numbers were lower in urban than in forest-reared broods. Thus, at least in our comparison of two sites, the hard labour of urban parents did not fully pay off, suggesting that improved habitat management is required to support urban-breeding birds.

**Electronic supplementary material:**

The online version of this article (10.1007/s00442-020-04678-w) contains supplementary material, which is available to authorized users.

## Introduction

Urbanisation is rapidly transforming natural habitats through spatial fragmentation (McDonald et al. 2013), altered climate (Grimmond 2007), increased pollution (Isaksson 2015), and altered vegetation and associated biotic composition (Narango et al. 2018). In their response to this novel environment, species are polarised between a small number of winners (exploiters) and greater numbers that to some degree adjust to (adapters) or flee (avoiders) urban environments (McKinney 2002). The general trend is a decrease in species richness as urbanisation intensifies (Sol et al. 2014; Batáry et al. 2018), calling for a deeper understanding of the mechanisms driving a species’ success in urban environments.

In-depth studies of the ecology and fitness of urban fauna often focus on birds because they are easily encountered in cities (e.g. Chamberlain et al. 2009; Isaksson 2015; Glądalski et al. 2017; Narango et al. 2018; Pollock et al. 2017; Seress et al. 2018). Urban adapters are of particular interest for efforts to counteract biodiversity loss because populations in urban areas often have lower reproductive success than those in more natural environments (e.g. smaller clutch size, more nest failures and lower nestling weight; Mennechez and Clergeau 2006; Chamberlain et al. 2009; Seress et al. 2012; Pollock et al. 2017). Cities could thus be “ecological traps” (Robertson and Hutto 2006) and function as sinks for some species that persist in urban sites for apparent benefits, such as access to feeders or nest sites (Battin 2004; Sumasgutner et al. 2014; Pollock et al. 2017). Identifying the drivers of reproductive success in urban birds could allow for targeted management of urban environments to counteract such negative effects.

Here we investigated season-dependent dietary requirements as one potential constraint on reproductive outcomes in an urban adapter, the blue tit, *Cyanistes caeruleus* (Pollock et al. 2017). During winter, when resources are scarce in the wild, cities may appear favourable for birds due to food provided by human activity, whereas during the breeding season cities may lack sufficient high-quality resources for raising offspring (e.g. micronutrients such as carotenoids and essential aminoacids which are available from caterpillars and spiders; Ramsay and Houston 2003; Eeva et al. 2010; Demeyrier et al. 2017). Breeding success in urban birds could be limited by reproductive output (clutch size), nest success, or offspring quality (e.g. fledgling body mass), reducing the number of surviving and recruiting young. Reduced reproductive outcomes could arise for several reasons: first, through unmet specialist dietary needs of chicks (Mennechez and Clergeau 2006; Eeva et al. 2010; García-Navas et al. 2013b); second, through higher search effort for suitable food (Naef-Daenzer and Keller 1999; Tremblay et al. 2004; Stauss et al. 2005; Staggenborg et al. 2017); and third, through impaired health and poor performance of urban parents (Isaksson 2015; Capilla-Lasheras et al. 2017; Ibáñez-Álamo et al. 2018). These factors can act in combinations. For example, when parents have to work hard to source suitable food, they might shift to lower-quality diet (Tinbergen 2002; Wright et al. 2002), reduce provisioning (Naef-Daenzer and Keller 1999; Staggenborg et al. 2017), or suffer decreases in condition and survival prospects (Thomas et al. 2001).

The hypothesis that urban birds with specialist needs for chick rearing are limited by resources is supported by studies of species that specialise on provisioning nutritious arthropod diets (particularly songbirds in the parid family: blue tits, great tits *Parus major,* and Carolina chickadees *Poecile carolinensis*; Glądalski et al. 2017; Narango et al. 2018; Pollock et al. 2017; Seress et al. 2018). Parids raise very large clutches by exploiting a short, sharp spring peak in caterpillar availability. Caterpillars are easily ingestible for nestlings and are particularly rich in nutrients, such as carotenoids (Bañbura et al. 1999; Eeva et al. 2010). Parids may thus suffer decreased reproductive success when they cannot fully capture the caterpillar peak (Visser et al. 2006), at least in managed forests (Wesołowski and Rowiński 2014). Due to lower native tree abundance, availability of caterpillars is lower in urban than in forest habitats, and chick provisioning with caterpillars is also lower, making the scarcity of this preferred feeding source the most likely contributor to frequently low urban reproductive success (Glądalski et al. 2017; Pollock et al. 2017; Narango et al. 2018; Seress et al. 2018; but see Isaksson and Andersson 2007).

However, there are still important gaps in the understanding of the critical link between food availability and reproductive outcomes, in particular relating to parental compensation of food shortages in urban habitats. First, parents can partly offset local shortages of preferred diets in poor habitats by increased search effort (Naef-Daenzer and Keller 1999; Tremblay et al. 2004; Stauss et al. 2005; Staggenborg et al. 2017). Some studies estimated higher nest provisioning rates in urban birds (Pollock et al. 2017), but total workload will depend also on the distance covered by birds (Tinbergen 2002; Wright et al. 2002). Reduced flight distances in urban birds could be expected due to poor condition (Isaksson 2015; Capilla-Lasheras et al. 2017; Ibáñez-Álamo et al. 2018). As such it remains unclear whether urban parents indeed increase their efforts for chick provisioning (Glądalski et al. 2017; Pollock et al. 2017; Seress et al. 2018).

Second, parents can partly offset a lack of preferred diet items by provisioning alternative food items in the city, such as invertebrates with insufficient nutritional value or anthropogenic foods (Shawkey et al. 2004; Mennechez and Clergeau 2006; García-Navas et al. 2013a). Anthropogenic foods in particular may be unsuitable or even cause chick mortality (Pollock et al. 2017). However, the use of alternative foods for chick provisioning in cities is poorly understood. Our knowledge is mainly based on visual observations, which provide limited information because delivered food items can only be coarsely identified and categorised (Seress et al. 2012; Samplonius et al. 2016; Pollock et al. 2017). For example, visual observation could easily fail to distinguish anthropogenic foods from natural foods, for instance, mealworms from caterpillars (CJ, personal observation).

When linking reduced reproductive outcomes to diet quality, it is therefore essential to quantify parental effort in feeding young, and to comprehensively characterise provisioned food. These objectives can now be addressed by advances in animal tracking and high-throughput sequencing. First, tracking studies can provide detailed information on behaviour. For example, using telemetry, Tremblay et al. (2004) showed that blue tits in a caterpillar-poor, semi-natural forest environment increased their foraging efforts. By doubling their foraging distance, parents were able to deliver caterpillar biomass similar to that of parents in a caterpillar-rich environment. For interpreting such findings, an important aspect is quantification of tree density because availability of deciduous trees, in particular oak (*Quercus* sp.), determines the distribution of caterpillars in the environment (Wint 1983; Perrins 1991; Pulido and Díaz 1997; Wilkin et al. 2009). Second, songbird diets can be studied in fine resolution via recently developed faecal DNA metabarcoding (Trevelline et al. 2016). This technique has enormous potential: from each faecal sample, dozens of unique prey taxa can be non-invasively identified (Jedlicka et al. 2013; Crisol-Martínez et al. 2016; Trevelline et al. 2016). Diet metabarcoding can provide much greater taxonomic resolution than video footage, allowing us to distinguish between items that are morphologically similar yet have very distinct ecological implications. Faecal metabarcoding may also be able to provide information on secondary consumption (Sheppard et al. 2005; Bowser et al. 2013; Roslin and Majaneva 2016): plant material in the nestling diet, potentially consumed by herbivorous prey, may provide information about additional links in the food web.

Here, we combined animal tracking, metabarcoding, and habitat and nestbox monitoring to establish links between the urban chick-rearing environment and reproductive outcomes. Due to the multi-layer, integrated approach of this study, we were able to consider only limited sample sizes of blue tits, measured at only 1 urban and 1 forest site. We acknowledge that our results may thus not necessarily be generalisable to all urban habitats or species. However, we were able to build upon the detailed knowledge of the local urban and forest blue tit populations, including monitoring of provisioning and of reproductive success (Jarrett et al. 2017; Pollock et al. 2017; Capilla-Lasheras et al. 2017). Specifically, we tested the following predictions: (a) urban birds will fly further afield to provision their young; (b) despite increased foraging effort, the diet delivered to the chicks in the city will contain fewer caterpillars but a wider range of foods overall, including items from anthropogenic sources; and (c) reproductive outcomes will be reduced in the city, indicating that the hard labour of urban parents does not fully compensate for the poor environment.

## Materials and methods

### Field data collection and information processing

*Field sites* (see Supplementary Fig. 1): From April to June of 2016, we compared habitat characteristics and breeding biology of blue tits breeding in woodcrete nestboxes at the city and forest sites. City blue tits bred in 40 nestboxes in Kelvingrove Park in Glasgow (55°52′ N, 4°17′ W; 71 total nestboxes). Kelvingrove Park is an urban green space along the river Kelvin, consisting of managed lawns, unmanaged riverbank vegetation, sports areas, and trees. Trees are mostly scattered or in stands, and consist of a mix of native and introduced species including low proportions of oak and birch (*Betula* spp.). Forest blue tits bred in 124 nestboxes in mixed deciduous, oak-dominated woodland surrounding the Scottish Centre for Ecology and the Natural Environment, on Loch Lomond, Scotland (56°7.5′ N, 4°37′ W; 280 total nestboxes; Pollock et al. 2017; Supplementary Methods).

*Avian fieldwork* (see Supplementary Methods): Starting on 14th April, we recorded nest building and egg laying weekly across all nestboxes, and we calculated the earliest possible hatch date based on date of clutch completion (see Jarrett et al. 2017). From the estimated hatch date onwards we checked nests every second day until hatching to precisely age broods. After hatching, we resumed weekly monitoring. During these visits, females that were present in the box were gently removed from nests and then placed back once we had finished inspecting. We quantified the following reproductive outcomes: clutch size, number of hatchlings and fledglings, hatching success (hatchlings/eggs), fledging success (fledglings/hatchlings), and fledging body mass. Fledging body mass was inferred from pre-fledging mass of nestlings on post-hatching day 13 (where hatching day = day 0). Inferring fledging mass from 2-week old tits is conventional, as body mass growth has levelled off (Kunz and Ekman 2000) and nest controls are still safe, whereas disturbing older chicks becomes hazardous for their lives (Naef-Daenzer and Keller 1999).

For the in-depth study, we chose 8 focal nestboxes containing blue tit broods at each site according to their suitability for telemetry and their logistical feasibility (henceforth “tracked broods”). However, one brood in the city died at day 7 of nestlings’ lives; for this brood, we did not collect nestling mass data, faecal samples, or video footage (described below). The mean hatch date for tracked broods was 16 ± 7 May in the city and 24 ± 3 May in the forest, whereas mean hatch dates for the remaining broods was 21 ± 7 May in the city and 24 ± 5 May in the forest. We caught one of the parents from each brood on post-hatching day 4–6 while it provisioned its brood. We caught 5 females and 3 males in the forest, and 3 females and 5 males in the city. The adult bird was equipped with a radio transmitter (PIP31; Biotrack, Dorset, UK; 0.35 g) via eyelash adhesive and a small amount of superglue as described in Nord et al. (2016). We recorded two 24 h periods of parental provisioning from within each nestbox, by installing infrared camera systems on post-hatching days 7 and 11 (Pollock et al. 2017). After each 24 h period, cameras were taken down. On post-hatching day 13, we weighed and ringed all nestlings. We collected faecal samples from nestlings directly into vials containing 100% ethanol by holding the vial below the cloaca of the nestling. We aimed to collect faecal samples from at least two hatchlings per nest and achieved this for 13 nests (6 in the forest and 7 in the city). For 2 nests, we collected just 1 sample, and we did not collect any faecal samples from the failed brood. All samples were stored at − 20 °C during the field season.

*Telemetry* (see Supplementary Methods): After tagging the adult birds with radio transmitters, we left them to habituate for a period of approximately 24 h (city: 28.0 ± 4.1 h; in the forest: 29.5 ± 14.3 h). Then, we tracked birds with Lotek SRX400 receivers and Yagi antennas. Two observers (CJ and HM), standing at least 15 m away from the nestbox at a 90° angle, triangulated the position of the bird, taking compass bearings every 2 min over 30-min tracking periods. We scored signal quality of each position fix (“good”, “moving” or “bad”; see Supplementary Methods), and excluded all fixes classed as “bad” from analysis; there were more “bad” fixes in the city than in the forest (45 and 26 respectively), likely due to interference with buildings. We recorded 3–5 tracking periods of 30 min per bird, collected over 1–4 days when the nestlings were 6–11 days old (fixes: total 666, after data clean-up 570; city: *n* = 303; forest: *n* = 267). The number of fixes per bird ranged from 13 to 58, spread across the day. We calculated bird locations from triangulation using the Sigloc package (Berg 2015) within R 3.3.1, and foraging distances (distance between nestbox and each bird location) using the package Geosphere (Hijmans et al. 2012).

*Video recording of parental provisioning* (see Supplementary Methods): To estimate provisioning items and rates, we aimed to extract 4 half-hour periods of footage per tracked brood using VideoLAN VLC (8:00–8:30 and 19:00–19:30 per sampling day, henceforth “morning” and “evening”, following Pollock et al. 2017). On several occasions, we were unable to record footage due to technical failures; our final dataset consisted of 23 periods at each site covering 7 nestboxes. We calculated provisioning rate as the number of parental entries per half-hour. We identified items delivered by parents as either caterpillars or other invertebrates and calculated their relative abundance at each nestbox; non-identified items (16%) were excluded. We calculated the volume of caterpillars delivered using the formula (*π*/4) × *L* ×*W*^2^ (Blondel et al. 1991), where total length (*L*) and mean width (*W*) were estimated using the diameter (32 mm) of the nest hole as a reference. We calculated caterpillar biomass as the total caterpillar volume delivered to the nest in half an hour.

*Tree sampling* (see Supplementary Methods): We calculated tree density and numbers of oaks and birches in each habitat in a 35 m radius around the 16 focal broods used for radio telemetry. The radius represents the average foraging trip calculated from telemetry results (34.3 m, see below).

### Metabarcoding and bioinformatics

DNA was successfully extracted from 26 faecal samples using a magnetic bead protocol modified from Vo and Jedlicka (2014) with the following modifications: we utilised 0.05 g faecal matter (wet weight); samples were homogenised in a BeadBeater (BioSpec Products) for 3 cycles of 30 s with a 30-s pause between.

Triplicate PCR of each sample was performed targeting two loci (see Supplementary Methods): (1) for arthropod diet items, an approximately 200 bp portion (without primers) of the cytochrome oxidase I (COI) gene was amplified using the ZBJ primers from Zeale et al. (2011); (2) for plant diet items, a portion of the rbcL gene was amplified using custom designed primers (rbcL3/rbcL4 was 90 bp, rbcL5/rbcL6 was 110 bp, and rbcL7/rbcl8 was 140 bp without primers, Supplementary methods). A sufficient number of reads was obtained only for the rbcL3/rbcL4 primer set. Primers were modified to contain a portion of the Illumina adapter sequence (Supplementary Table 1). PCR primers are generally assumed to be universal, but all have some taxonomic biases. The ZBJ primers amplify Dipteran and Lepidopteran taxa particularly well and may be less successful for other arthropod orders (Clarke et al. 2014). Here, we are performing a comparative analysis, so any primer bias present should impact results for both populations to the same extent, e.g. the primers should amplify Lepidopterans particularly well, regardless whether they occur in the diet of city or forest birds.

For each sample, the triplicate PCR products were pooled for each locus in equal volumes and then 7.5 µL for the COI pool and 2.5 µL of the rbcL pool were combined. Samples were cleaned using 0.8 × carboxyl paramagnetic beads, following the protocol stated by Rohland and Reich (2012) using 80% ethanol for washes. A second PCR was conducted using primers complementary to the overhang sequence and containing an individual specific pair of indices (Supplementary Methods). Samples were then cleaned using 0.8 × carboxyl paramagnetic beads as above, quantified, pooled, and sequenced on the Illumina MiSeq platform to produce 150 bp paired-end sequences.

Raw sequences were trimmed and error corrected following Schirmer et al. 2015 (Supplementary Methods) and then merged. Data for each primer set were split using a custom python script, and PCR primers were trimmed off. For the COI dataset, non-target sequences (e.g. those potentially belonging to the birds or humans) were filtered out using BLAST. The data were filtered for potential chimeric sequences and then clustered into molecular operational taxonomic units (OTUs) at the 97% identity level using Sumaclust (Mercier et al. 2013). Following Alberdi et al. (2018) and Aizpurua et al. (2018), we assigned taxonomy via a BLAST search of the Genbank NT database. Taxonomy was assigned to each OTU based on identity: For matches with ≥ 95% identity we assigned order-level taxonomy; for ≥ 96.5%, we assigned family level, and for ≥ 98% we assigned genus and species-level taxonomy.

### Statistical analysis

Statistical analyses were conducted in R 3.3.3 (R Core Team 2019). All linear mixed models (Supplementary Table 2) were fit in the package lme4 (Bates et al. 2015), whereas we used the MASS and STATS packages for linear and general linear models. Assumptions of normality of residuals and homogeneity of variance were checked by inspecting residuals plots. We constructed models containing explanatory variables chosen a priori based on the literature and our knowledge of the system variables. We chose the following starting models (Supplementary Table 2): Tree density was analysed for site only and OTUs from faecal metabarcoding were analysed for site and date and the interaction between these two (including nestbox as random effect). Provisioning rates, and proportions and volumes of provisioned items, were also analysed by site and date, with nestbox as random effect, and additionally by time of day and nestling age. Total biomass delivered (volume per 30 min) was analysed similarly by site and time with nestbox as random effect, but additionally by the interaction between site and foraging distance. Foraging distance was analysed by site, time of day, sex, nestling age, surrounding tree density, and brood size in interaction with site, with nestbox as random effect. All variables of nest success were tested for effects of site and date. Fledgling body mass was analysed by site, brood size, and hatch date, and in a separate model, by provisioned caterpillars, brood size, and hatch date, with nestbox as random factor. Adult body mass was analysed by site and sex. Full models containing dates as explanatory variable included both the quadratic and the linear forms.

We modelled count data for tree abundance using Generalised Linear Models with a Negative Binomial error structure (Supplementary Table 2). Differences between sites in all aspects of diet and foraging distance were investigated by linear mixed models with a Gaussian error structure. We compared life-history data between sites using Generalised Linear Models: clutch size and number of fledglings with a Poisson error structure and hatching and fledging success with a Binomial error structure. The latter was used because hatching and fledging success were calculated as proportions. We report reproductive outcomes for the 130 non-focal broods in our urban and rural study sites and for the 15 tracked broods used for radio telemetry and metabarcoding (excluding the failed brood).

We performed Likelihood Ratio Tests of fully nested models (LRTs; cut-off probability *P* > 0.05) to eliminate non-significant variables. We then used minimal adequate models to estimate coefficients. However, in all models we retained the site covariate to quantify effect sizes and control for unaccounted differences between forest and city sites (presented in Supplementary Table 3). We arrived at the same minimal adequate models comparing candidate models with LRTs and Akaike’s Information Criteria (AICc; cut-off = ∆AICc > 2 from best-fit model). Throughout the results, we report mean and standard deviation as summary statistics (mean ± SD). We report the difference in Log Likelihood between models as Chi-squared values (*χ*^2^) with associated *p* values. The difference in degrees of freedom between models was always 1. For the estimate and error of individual parameters within each model, one should refer to Supplementary Table 3. We also report the sample size for each set of models; if the sample size is not mentioned, it is the same as the previous model.

## Results

### Tree community composition

The forest had 3 times more trees than the city (*n* = 16, *χ*^2^ = 15.2, *P* < 0.001; Supplementary Table 3.a), and 30 times more oaks (*χ*^2^ = 597.0, *P* < 0.001; Supplementary Fig. 2). The number of birches was also 5 times higher in the forest (*χ*^2^ = 7.0, *P* = 0.01). The city site contained more trees that were neither oaks nor birches (*χ*^2^ = 10.2, *P* = 0.001), which mostly represented non-native species such as sycamore (*Acer pseudoplatanus*).

### Foraging distance

**Fig. 1:**
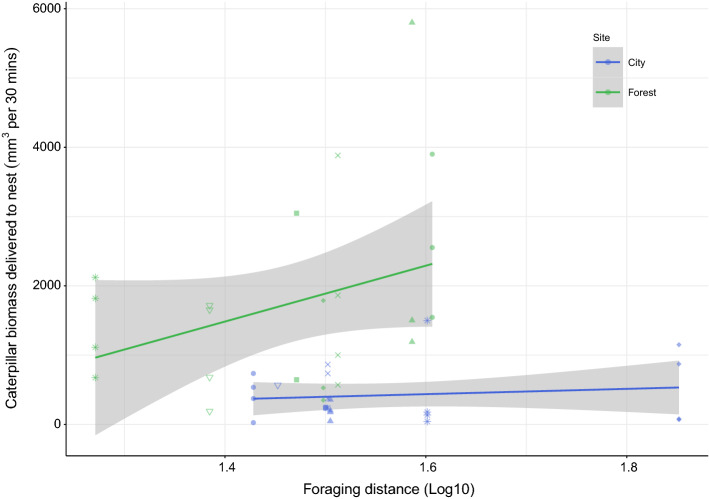
The effect of blue tit foraging distance on the biomass of caterpillars delivered to the nests in the forest (green) and the city (blue). The *x*-axis shows foraging distance (m), averaged for each nestbox and log transformed. Each point on the *y*-axis represents the total caterpillar biomass delivered to a given nestbox during each of the 30-min video observation periods. Final sample sizes were as follows: *n* = 23 in the city (5 broods with 4 periods, 1 brood with 2 periods, 1 brood with 1 period, and 1 brood with 0 periods) and *n* = 23 in the forest (3 broods with 4 periods, 3 broods with 3 periods, 1 brood with 2 periods, and 1 brood with 0 periods). Therefore, several points on the y-axis are plotted against the same foraging distance as they correspond to the same nestbox; it is noted that we have added jitter (using ggplot2; Wickham 2016) to foraging distance for visibility

The variables significantly affecting foraging distance were site, sex, number of hatchlings, and age of nestlings (*n* = 570; Supplementary Table 3.b). In the forest, mean foraging distance was 30.6 ± 19.2 m, and foraging trips exceeding 50 m comprised 13% of trips. In the city, parents flew further: mean foraging distance was 39.2 ± 23.7 m, and in 24% of trips distances exceeded 50 m (Fig. 1). Foraging distance was higher in males and increased with number of hatchlings and age of nestlings.

### Video-recorded parental provisioning

Provisioning rates per 30 min at the two sites differed neither per nest nor per nestling (*n* = 57, per chick, city: 2.90 ± 1.49, forest: 2.63 ± 1.34; per nest, city: 22.13 ± 11.16, forest: 21.70 ± 10.16 in forest, *P* = 0.70 for both measures; Supplementary Table 3.c). Caterpillars were delivered in 73 ± 16% of visits by parents in the forest but only in 31 ± 9% of visits in the city (*χ*^2^ = 20.0, *P* < 0.001; Supplementary Fig. 3). Additionally, the average volume of individual caterpillars in the forest was significantly larger than that in the city (114.8 ± 28.8 mm^3^ and 71.1 ± 33.8 mm^3^, respectively; *χ*^2^ = 7.2, *P* < 0.007). The proportion of visits during which non-caterpillar arthropods were delivered to the nest was significantly lower in the forest than in the city (12 ± 12% and 39 ± 13% respectively; *χ*^2^ = 11.8, *P* < 0.001).

The effect of parental foraging distance on delivered caterpillar biomass differed between sites (*n* = 57, *χ*^2^ = 5.9, *P* = 0.01; Fig. 1). In the forest, increasing foraging distance was rewarded with higher caterpillar yield. For example, increased foraging distance from 20 to 40 m resulted in 140% more caterpillar biomass (from 1066.5 ± 294.7 to 2409.7 ± 290.1 mm^3^). In the city, the distance foraged by parents did not affect caterpillar biomass delivered; in other words, city birds travelling further did not produce more caterpillar biomass for their young.

### Faecal metabarcoding

Of the 26 chick faecal samples we extracted, we successfully amplified DNA from 17, comprising 7 forest samples (from 6 broods) and 10 urban samples (from 7 broods). We identified 211 arthropod OTUs (Supplementary Table 4). Of these OTUs, we identified 32.2% to species level, and 90.5% to order level. The mean number of OTUs per sample was 29.8 ± 20 taxa.

**Fig. 2:**
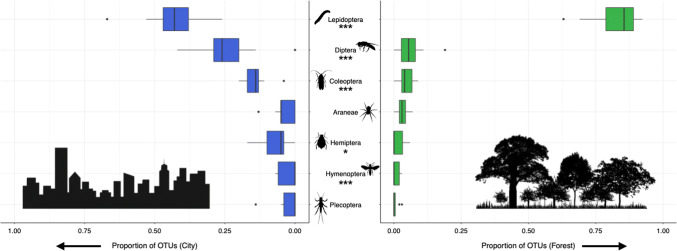
Proportion of OTUs per sample from each arthropod order present, at the city (blue) and forest (green) sites. The bold line within each box indicates the median value; the lower and upper limits of the boxes represent the second and third quartiles, respectively; and the lines extend to the farthest outliers within 1.5 times the interquartile range. Orientation of x-axes for city and forest sites is reversed between sites

The proportion of OTUs per sample from the order Lepidoptera was significantly higher in the forest than in the city (*n* = 17, *χ*^2^ = 26.0, *P* < 0.001; Supplementary Table 3.d). In the forest, Lepidoptera comprised 82 ± 11% of all OTUs, and in the city 44 ± 10% (Fig. 2). The proportions of OTUs from the orders Diptera (*χ*^2^ = 13.0, *P* < 0.001), Coleoptera (*χ*^2^ = 15.2, *P* < 0.001), Hemiptera (*χ*^2^ = 5.4, *P* = 0.02), and Hymenoptera (*χ*^2^ = 17.6, *P* < 0.001) were significantly higher in the city than in the forest. The proportions of some of these orders were also affected by date. All other orders did not differ significantly between sites or dates.

While the proportion of OTU reads obtained from diet metabarcoding may not perfectly reflect the mass of items in the diet, there is some evidence of a rough correlation between the two such that the rank order of diet items is preserved (Deagle et al. 2010; Bowles et al. 2011; Srivathsan et al. 2015). Therefore, we pooled OTUs by site and ranked those with taxonomic assignments by total number of reads (highest number of reads = rank 1). For forest samples, ranks 1–10 were all OTUs from the order Lepidoptera (Table 1), and ranks 1–4 were comprised exclusively by Lepidopterans of the family Geometridae. City samples showed a wider range of arthropod orders in ranks 1–10 (Diptera, Coleoptera, Lepidoptera, Araneae, and Hemiptera), but ranks 1 and 2 were taken up by Diptera of the family Syrphidae (hoverflies). Of particular interest, the OTU ranked 7th most abundant in the city samples belongs to the mealworm *(Tenebrio molitor).*

**Table 1:** Arthropod taxa OTUs from faecal metabarcoding of city and forest blue tit nestlings

Rank	Reads	Order	Family	Genus	Species	
1	12,131	Diptera	Syrphidae	Syrphus	*S. torvus*	City
2	8290	Diptera	Syrphidae	Syrphus	Unassigned	
3	8166	Lepidoptera	Noctuidae	Cosmia	*C. trapezina*	
4	5135	Lepidoptera	Tortricidae	Hedya	*H. nubiferana*	
5	3031	Lepidoptera	Geometridae	Apocheima	*A. pilosaria*	
6	2259	Diptera	Syrphidae	Unassigned	Unassigned	
7	505	Coleoptera	Tenebrionidae	Tenebrio	*T. molitor*	
8	220	Hemiptera	Aphididae	Drepanosiphum	*D. platanoidis*	
9	197	Araneae	Philodromidae	Philodromus	Unassigned	
10	163	Lepidoptera	Tortricidae	Ptycholoma	*P. lecheana*	
1	25,091	Lepidoptera	Geometridae	Hydriomena	*H. furcata*	Forest
2	18,389	Lepidoptera	Geometridae	Operophtera	*O. brumata*	
3	6019	Lepidoptera	Geometridae	Operophtera	*O. fagata*	
4	4310	Lepidoptera	Geometridae	Erannis	*E. defoliaria*	
5	4227	Lepidoptera	Noctuidae	Cosmia	*C. trapezina*	
6	4083	Lepidoptera	Geometridae	Agriopis	*A. leucophaearia*	
7	3702	Lepidoptera	Noctuidae	Brachylomia	*B. viminalis*	
8	1401	Lepidoptera	Geometridae	Apocheima	*A. pilosaria*	
9	1140	Lepidoptera	Ypsolophidae	Ypsolopha	*Y. ustella*	
10	920	Lepidoptera	Tortricidae	Acleris	*A. rhombana*	

Shown are ranks 1–10 by number of reads (highest number of reads = rank 1) for city and forest faecal samples

In addition to arthropods, chick faecal samples contained 35 plant OTUs, 25 of which were identified to order level (Supplementary Table 4). The samples contained 16 distinct plant orders, the majority of which (11) were found only in samples from the city. Four orders (Fabales, Fagales, Rosales, and Sapindales) occurred in samples from both environments, and one order (Myrtales) occurred only in those from the forest. The order Fagales, which includes oak and birch, was much more frequent in the forest (48 ± 24% of OTUs) than in the city (17 ± 12%; *n* = 17, *χ*^2^ = 10.0, *P* = 0.001).

### Reproductive outcomes

**Fig. 3:**
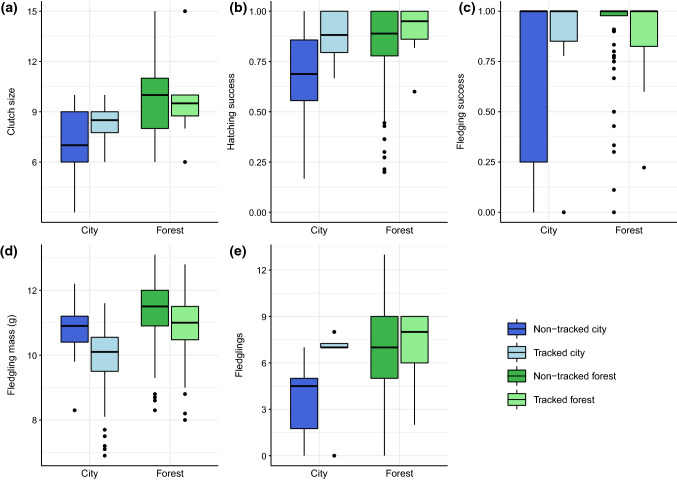
Breeding outcomes at the city (blue) and forest (green) sites. **a** Clutch size, **b** hatching success, **c** fledging success, **d** fledgling body mass, and **e** number of fledglings. Darker colours represent the non-tracked broods (*n* = 130), and lighter colours represent tracked broods (i.e. those used for telemetry and provisioning data; *n* = 16). The bold line within each box indicates the median value; the lower and upper limits of the boxes represent the second and third quartiles, respectively; and the lines extend to the farthest outliers within 1.5 times the interquartile range

Clutch size in non-tracked boxes was larger in the forest by 2.0 eggs (*n* = 130, *χ*^2^ = 6.6, *P* = 0.01; Fig. 3; Supplementary Table 3.e), and number of fledglings higher by 2.9 chicks (*χ*^2^ = 7.6, *P* < 0.001). Hatching success and fledging success were marginally higher in the forest (*P* > 0.05; Fig. 3). Fledgling mass in the forest was 11.3 ± 0.7 g and in the city 10.8 ± 0.7 g (*n* = 129, *χ*^2^ = 2.4, *P* = 0.12; Fig. 3).

When considering tracked boxes, clutch size was higher in the forest by 1.3 eggs (*n* = 16, *χ*^2^ = 6.9, *P* = 0.4; Fig. 3; Supplementary Table 3.f) and number of fledglings was higher by 0.8 chicks (n = 16, *χ*^2^ = 0.33, p = 0.56) although differences were not significant. Hatching success and fledging success were also marginally higher in the forest than the city (*P* > 0.05; Fig. 3). The clearest difference was in fledgling mass, which was significantly higher in the forest (forest: 10.9 ± 0.9 g, city 9.9 ± 1.1 g, *n* = 120*, χ*^2^ = 16.1, *P* < 0.001; Fig. 3). We also detected quadratic effects of date on fledgling mass, with a peak in mid-May. Conversely, site had no significant effect on parent body mass (*P* > 0.05).

Direct links between fledgling body mass and the provisioned proportion of caterpillars (video estimates) were partly supported (Supplementary Table 3). Proportion of caterpillars was retained in the best-fit model to explain fledgling body mass (*n* = 111, *χ*^2^ = 4.6, df = 1, *P* = 0.03), but the effect size was non-significant (*P* = 0.23).

## Discussion

We found that blue tit parents in an urban environment increased their foraging effort compared to their forest conspecifics, but still provisioned their chicks with strikingly different food items, lacking critical caterpillars. The low-quality diet provisioned to chicks in the city likely contributed to the lower body mass of chicks in the urban broods.

As we predicted, the density of oaks was far lower in the city than in the forest. Tree community composition in the city likely affected insects, especially taxa, such as caterpillars, that depend heavily on oaks (Wint 1983). Indeed, Pollock et al. (2017) found that in our study system, the forest site contained up to 10 times the abundance of caterpillars of the urban site. The forest site also contained higher numbers of Arachnida, whereas at the urban site, Hemiptera (in particular aphids) were far more abundant (Pollock et al. 2017). Qualitatively similar differences were confirmed anecdotally also for the current study year, but the low sample sizes did not allow robust analyses (Jarrett et al., unpublished data). Our data add further evidence of poor representation of native trees in urban habitats compared to forest habitats, with likely knock-on effect on invertebrate communities (Glądalski et al. 2017; Pollock et al. 2017; Narango et al. 2018; Seress et al. 2018; but see Isaksson and Andersson 2007). A shortage of insects of the given taxa could alternatively, or in addition, be caused by other features of the urban environment, for example, chemical or light pollution (Isaksson 2015; Owens and Lewis 2018).

During the breeding season, blue tits are highly selective and prefer to provision their nestlings with caterpillars, which have high nutrient content and can be rapidly consumed (Bañbura et al. 1999; Eeva et al. 2010). Hence, as expected from studies of more natural habitats with varying caterpillar availabilities (Tremblay et al. 2004; Stauss et al. 2005), urban blue tits in our study worked harder at foraging than our forest blue tits. Although provisioning rates were similar at both sites, both per nest and per nestling, blue tit parents in the city flew further to collect food. It is possible that blue tits extended their flight distance to reach trees that provided rich nestling diet (Hinsley et al. 2008), as other studies have shown that parids actively select such trees (Narango et al. 2017). Based on our data, urban parents would have spent more energy on foraging trips (Hinsley et al. 2008) and will have had less time for self-maintenance or brooding than parents in the forest. However, there was no direct reward for the increased flight distances of urban birds: in contrast to the forest habitat, flying further afield in the city was not associated with a discernible increase in provisioned caterpillar biomass. Interestingly, differences in foraging distance between the city and the forest were smaller than differences between habitat types described in other studies (Tremblay et al. 2004). It is possible that urban birds responded to the low pay-off of increased foraging effort directly by no further increases in flight distance.

In the forest site, caterpillars constituted the major food source (73% of delivered items, 82% of OTUs), while in the city they were significantly less frequent (31% of delivered items, 44% of OTUs). Urban parents compensated for the shortage of caterpillars by provisioning more non-Lepidopteran invertebrates than forest parents, as evident from both faecal metabarcoding and video footage analysis. Although some items, such as spiders, can be beneficial for nestlings (Ramsay and Houston 2003; Samplonius et al. 2016), items, such as crane flies and aphids, delivered frequently in the city, may provide limited nutrition (Eeva et al. 2010). The metabarcoding provided higher-resolution evidence of Diptera, Coleoptera, and Hemiptera being consumed in significantly greater abundance by urban nestlings. Intriguingly, the top two urban ranks of OTUs were held by dipteran family Syrphidae, which as larvae typically specialize on aphid prey (Chadwick and Goode 1999). The availability of Syrphidae larvae in the city may thus be driven by the high abundance of aphids. Coleopteran mealworms are a likely anthropogenic food source as in the United Kingdom they are commonly provided in bird feeders (Orros and Fellowes 2015). Mealworms were abundant in city bird diets, and unexpectedly also in a low number of forest bird samples. These could have originated from bird feeders in gardens of interspersed cottages (within ca. 1.5 km from the study site). Furthermore, detection of the plant orders Asterales and Poales in the urban diet potentially represent provisioning of sunflower seeds and millet, respectively. Plant sequences from faecal metabarcoding also provided evidence for the link between caterpillars and oak trees; the order Fagales comprised 48% of all plant OTUs in the forest, yet only 17% in the city.

The differences between sites, most probably due to the available caterpillar biomass, affected reproductive outcomes. Clutch size was smaller in the city by 20%. Blue tits are limited by energy when raising their large broods (Thomas et al. 2001); therefore, parents could have reduced clutch size strategically or because of poor health. Adult blue tits at our urban site in 2015 showed elevated expression of immune genes (Capilla-Lasheras et al. 2017), and reduced immune function and elevated corticosterone levels have been reported from other urban sites (Watson et al. 2017). Given their smaller clutch sizes and apparent compensatory efforts, urban parents in our study were only slightly less successful at raising the broods until fledging. However, urban nestlings had lower pre-fledging body mass, which in parids predicts reduced prospects of recruitment and survival (Both et al. 1999).

Our findings on reproductive outcomes may be a conservative estimate of the bitter fruits of the urban parents’ hard labour. The study season in 2016 was favourable for blue tits at our sites, compared to 2015 when urban parents fledged less than one chick per nest (mean number of fledglings in the city in 2015 was 0.38 ± 0.3 compared to 4.1 ± 2.6 in 2016; Capilla-Lasheras et al. 2017; Pollock et al. 2017). An increasing number of studies, including our own, have reported that under severe weather conditions, urban birds suffer far greater loss of reproductive success than those in forest areas (Glądalski et al. 2017; Pollock et al. 2017; but see Whitehouse et al. 2013). Under more stressful environmental conditions, such as those of 2015, urban birds might further increase their parental effort while being even less able to compensate for features of the urban environment that are hostile to developing chicks (Salmón et al. 2016, 2018; Pollock et al. 2017). Therefore, at least under inclement breeding conditions, cities may well function as population sinks for apparently urban-adapting species. Long-term studies on urban populations with more robust sample sizes are needed to fully understand the implications of inter-annual variation in environmental conditions. Sample sizes and number of sites in our study were chosen to enable an in-depth, integrative approach for linking behaviour and ecology to high throughput dietary data. Although we acknowledge that this prioritisation carries risks of generalising from low sample sizes, our findings confirmed to greatest extent our specific a priori hypotheses.

## Conclusions

We have documented that urban blue tit parents work harder than those in the forest, probably due to reduced availability of high-quality nestling food in the city habitat. However, on at least three levels, this hard labour did not pay off: longer foraging distances in the city did not yield significantly more caterpillars; the diet of urban chicks was substantially shifted to include alternative foods; and low pre-fledging mass of urban chicks predicts reduced chances of future reproduction.

An increasing body of evidence has shown that the biodiversity supported by urban green spaces is extremely variable, and depends heavily on size, connectivity, management, and many other site-specific characteristics (Lepczyk et al. 2017). To optimise urban habitat for biodiversity conservation, we must fully understand the challenges facing urban adapters, including the particular vulnerabilities of their seasonal life-cycle stages, and the mechanisms they adopt to prevail. An upcoming research challenge will thus be to gain an integrative view of how the multiple urban stressors interact to affect wildlife. Mitigation against urban impact on birds and their arthropod prey should also address several targets, such as reducing chemical and light pollution. Yet it could fruitfully begin with simple measures like planting native trees at higher densities in urban parks to encourage caterpillar populations and improve the breeding outcomes of passerines.

## Electronic supplementary material

Below is the link to the electronic supplementary material.

Supplementary file1 (PDF 362 kb)

## Data Availability

The data supporting the results, scripts and further information are available on Figshare 10.6084/m9.figshare.12444686.
