# Supplementary material for: Bitter fruits of hard labour: diet metabarcoding and telemetry reveal that urban songbirds travel further for lower-quality food
Source: Oecologia. 2020 Jun 13;193(2):377–88. doi: 10.1007/s00442-020-04678-w (PMC7320956; doi:10.1007/s00442-020-04678-w)
Supplement: Supplementary file 1 — Supplementary file1 (PDF 362 kb) [file 442_2020_4678_MOESM1_ESM.pdf]

Supplementary Material

Bitter fruits of hard labour: Diet metabarcoding and telemetry reveal that urban songbirds travel further for lower-quality food

Crinan Jarrett, Luke L. Powell, Heather McDevitt, Barbara Helm * and Andreanna J. Welch *

* shared corresponding authors: b.helm@rug.nl and a.j.welch@durham.ac.uk

Supplementary Methods

Field data collection and information processing

Field sites: Our nestbox set-up was located in the urban Kelvingrove Park in Glasgow (55°52' N, 4°17' W; 71 nestboxes) and in an oak-dominated deciduous woodland surrounding the Scottish Centre for Ecology and the Natural Environment (SCENE), on Loch Lomond, Scotland (56°7.5' N, 4°37' W; 280 nestboxes) (Suppl. Fig. 1). For details, see Pollock et al. (2017) and Møller et al. (2014). We used woodcrete boxes for parids (Schwegler, Germany) with an entrance hole diameter of 32 mm, hung on trees of heights between 150 – 350 cm.

Tree monitoring: We surveyed the numbers of oaks and of the second most common deciduous trees in our forest area, birch (*Betula* sp.) in a 35 m radius around the 16 focal nestboxes used for radio-telemetry (henceforth "tracked boxes"). A tape measure was used to measure 35m from the nestbox at intervals of 60º to each other, creating six sectors for ease of counting. All oak and birch trees (>0.5 m circumference at chest height) were identified and counted. All additional trees were counted and classed as "other". We also collected anecdotal data on invertebrate presence in 1 oak tree and 1 birch per site, as described by Pollock et al. 2017 (Suppl. Fig. 1).

Avian fieldwork: Starting on 14th April 2016, we recorded nest building and egg-laying weekly (see Capilla-Lasheras et al. 2017; Jarrett et al. 2017; Pollock et al. 2017). We calculated the earliest possible hatch date by estimating the date of the final egg (assuming one egg/day), and adding 12 days of incubation. From the estimated hatch date we checked nest boxes every second day until hatching occurred (mean hatch date: city = 21st May, forest = 24th May), distinguishing hatchlings from 1 day old chicks. Hatching day was counted as the first day in the birds' life (i.e. age = 0). Thereafter, remaining eggs and chicks were counted weekly and on day 13 chicks were measured and ringed. We refer to data from this day as "fledgling" data because nestlings by then are fully grown in body mass, and later sampling puts them at risk. To quantify reproductive outcomes, we calculated hatching success as the maximal numbers

of hatchlings/eggs, fledging success as the maximal numbers of fledglings/hatchlings, and used fledging mass (mass on post-hatching day 13) as a proxy for the probability of survival. After fledging, we recorded any dead chicks.

At each site, 8 focal nestboxes were chosen according to their suitability for telemetry, the ideal terrain being as even as possible, with a slight slope and some potential vantage points. One brood in the city died at day 7 of nestlings' lives; for this brood we did not collect nestling mass data, faecal samples or video footage. We caught one of the parents from each nestbox on the 5th (n=9), 6th (n=6) or 7th (n=1) day of the nestlings' lives while it provisioned its brood. The parents were tagged with a radiotransmitter (see below), and weighed, ringed, sexed and aged. Infrared camera systems were installed at each nestbox on day 7 and 11 of the nestlings' life (forest n=8, city n=8; custom-made systems by the University of Glasgow's Bioelectronics Unit; Pollock et al. 2017). On the 13th day of the nestlings' lives, we collected at least two faecal samples per nest. Samples were collected by picking up the chick and immediately holding a vial to its cloaca, in some occasions gently massaging the lower abdomen. Faecal samples were collected in vials containing 1.25ml of 100% ethanol and stored at -20°C during the field-season. Chicks were then weighed, ringed, and a blood-sample taken.

Telemetry: We tagged adults with radiotransmitters as described in Nord *et al.* 2016, using PIP31 single celled tag (Biotrack, Dorset, UK; weight 0.35g; maximal dimensions 7x7x4mm; battery type Ag337). Briefly, we trimmed feathers on the birds' lower back and attached a radiotransmitter using eyelash glue (Duo Eyelash Adhesive Clear White, waterproof) and a small amount of superglue. The birds were tracked over the next 1-4 days with Lotek wireless SRX400 receivers and Yagi antennas by two observers (CJ and HM). To triangulate the position of the bird, observers stood at an approximate 90º angle from the estimated bird location and took compass bearings in the estimated direction of the bird every 2 min, for periods of 30 min. The locations where the observers stood were recorded using GPS (Garmin, eTrex 30x). Whenever the bird was seen at any of the 2-min points during the tracking period, a bearing and an estimated distance from the observer were recorded. Observers coordinated their bearings with walkie-talkies, and synched watches. We carried out a total of 3-5 tracking periods of 30 min per bird, recording a total of 666 position fixings spread out across the day. For quality control of the data, the quality of the signal was recorded for each observation, based on the volume and stability of the signal. Categories used to indicate quality were "good" (when there was a steady volume signal with a clear direction), "moving" (when direction was

detectable but volume varied unexpectedly), and “bad” (when signal was faint and direction hard to detect). Any fixes where the signal quality was noted as being “bad” were excluded from analysis; there were more “bad” fixings in the city than the forest (45 and 26 respectively), likely due to interference with buildings. Triangulation calculations were carried out using the Sigloc package (Berg 2015) within R 3.3.1. Once all bird positions had been calculated, points were plotted using Google Earth (version 7.0.2.8415, 2012) and points deemed unlikely based on visual assessment of the bird’s position on the map were eliminated. The criteria used for exclusion were: if the point was a clear outlier compared to the majority of the data, or if the actual location of the bird was considered unrealistic (such as over water or behind rows of buildings). The number of position fixes after cleaning up the data was 570 (city: n=303; forest: n=267). We used the package Geosphere (Hijmans et al. 2012) to calculate foraging distances, considered as the distance (m) between the nestbox and each location of the bird, in a straight line.

Video recording of parental provisioning: Infrared camera systems were focused on the entrance hole from inside the nestbox and recorded for 24 hours, starting at approx. 5pm. Four standardised half-hour periods (8:00-8:30 and 19:00-19:30 per sampling day) were extracted for each nestbox using VideoLAN VLC Media Player. From counted parental entries, we calculated provisioning rate as the number of parental entries into the nestbox per half-hour period.

We classified provisioned prey items into two categories: caterpillars and non-lepidopteran invertebrates. Where possible we identified invertebrates to family level (e.g. aphids). We were unable to identify 16% (0.16±0.17) of all items delivered, with no bias for site (p>0.05). These items were discarded for analysis. For each half-hour period of footage, the number of items from each category delivered was divided by the total number of visits to obtain proportions of visits. The dimensions of the caterpillars delivered was estimated by obtaining total length *(L)* and mean width *(W)* using the diameter of the nest hole as a reference, and the formula $(\pi/4)*L*W^2$ (Blondel et al. 1991) to calculate the volume.

<u>Metabarcoding and bioinformatics</u>

PCR. PCRs were conducted in 15μL reactions containing 1X Multiplex PCR Mastermix (Qiagen), 0.13μM of each primer, and 3uL DNA extract. PCR thermocycling conditions followed an initial denaturation of 95°C for 5 min; 35 cycles of 95°C for 30 sec, 50°C for 1 min, and 72°C for 30 sec; and a final extension of 68°C for 10 min. For rbcL, multiplexed PCR

amplification was conducted by simultaneously using all three primer pairs with the same mastermix and thermal cycling protocols as described above. PCR was conducted up to seven times for each locus, with results examined on 2% agarose gels stained with ethidium bromide, until three positive PCR products were obtained.

Library construction and validation. After cleaning and pooling the PCR products as indicated in the main text, a dual indexing PCR was performed: 50μL reactions contained 1x HIFI Master Mix (KAPA), 0.5μM of each indexed primer, and 10uL bead cleaned PCR product. The thermal cycling conditions were: 72°C for 3 min; 98°C for 30 sec; 12 cycles of 98°C for 10 sec, 63°C for 30 sec, followed by 72°C for 3 min. Reactions were cleaned, quantified using the Qubit High Sensitivity DNA assay, and then pooled in equimolar ratios. The sequencing pool was analysed on a 2100 Bioanalyzer (Agilent Technologies) using the high sensitivity DNA kit. The final pool concentration was determined via quantitative PCR with the KAPA Library Quantitative Kit.

Bioinformatics. Unless otherwise specified, default parameters were employed. Raw sequences were analysed using FastQC (Andrews et al. 2011) and adapters were trimmed using CutAdapt v1.10 (Martin 2011). Following Schirmer et al. (2015), quality trimming was conducted using Sickle v1.33 (Joshi & Fass 2011) with the –x option to prevent 5' trimming (Joshi & Fass 2011). Then error correction was performed using BayesHammer (Nikolenko et al. 2013) through the SPAdes program v3.10.1 (Bankevich et al. 2012), and reads were merged using PEAR v0.9.6. (Zhang et al. 2014). Data for each primer set were split using a custom python script and PCR primers were trimmed off using CutAdapt. For the COI dataset, we filtered non-target sequences (e.g. those potentially belonging to the birds or humans) using BLAST: first, identical reads for each sample were collapsed using the obiuniq function in obitools (Boyer *et al.* 2016), while retaining the read counts. They were then queried against the curated COI Midori-LONGEST database (Machida et al. 2017) using blastn in BLAST+ v2.6.0. A custom python script was used to retain reads, and associated abundance information, for which the best match (based on bitscore) was classified as belonging to phylum Arthropoda, the alignment was ≥50% the length of the query sequence, and the e-value was $\leq 10^{-5}$. The data were then filtered for chimeric sequences using usearch v11.0.667 (Edgar 2010) implemented through a modified version of the program DAMe (Zepeda-Menoza et al. 2016) available at https://github.com/shyamsg/DAMe. The DAMe script convertToUSearch.py was used prepare

for OTU clustering, and to filter sequences ± 30bp of the target sequence length (without primers).

OTU clustering and taxonomy assignment. As discussed by Clare et al. (2011) and outlined by Razgour et al. (2011), we tested OTU clustering similarity thresholds from 94 – 100%, and used plots to identify the best threshold (97%) where over- and under-splitting were minimized (Krüger et al. 2014; Trevelline et al. 2016). We used the tabulateSumaclust.py script from the DAMe package to make OTU tables, retaining only OTUs with ≥ 5 sequences. To assign taxonomy, we used a BLAST search of the Genbank NT database: Using blastn in BLAST+ 2.7.1 we retrieved the best 20 matches for each representative OTU sequence. Using custom python and R scripts (Alberdi et al. 2018; Aizpurua et al. 2018), we retained the highest common taxonomic information among matches with the maximum bitscore, and assigned taxonomy to each OTU based on identity: For matches with ≥95% identity we assigned order-level taxonomy; ≥96.5% we assigned family-level, and for ≥98% we assigned genus and species-level taxonomy.

References

Aizpurua, O., Budinski, I., Georgiakakis, P., Gopalakrishnan, S., Ibañez, C., Mata, V. *et al.* (2018). Agriculture shapes the trophic niche of a bat preying on multiple pest arthropods across Europe: Evidence from DNA metabarcoding. *Mol. Ecol.*, 27, 815-825.

Alberdi, A., Aizpurua, O., Gilbert, M.T.P. & Bohmann, K. (2018). Scrutinizing key steps for reliable metabarcoding of environmental samples. *Methods Ecol.Evol.*, 9, 134-147.

Andrews, S., Lindenbaum, P., Howard, B. & Ewels, P. (2011). FastQC: a quality control tool for high throughput sequence data.

Bankevich, A., Nurk, S., Antipov, D., Gurevich, A.A., Dvorkin, M., Kulikov, A.S. *et al.* (2012). SPAdes: A New Genome Assembly Algorithm and Its Applications to Single-Cell Sequencing. *Journal of Computational Biology*, 19, 455-477.

Berg, S.S. (2015). The Package “sigloc” for the R Software: A Tool for Triangulating Transmitter Locations in Ground-Based Telemetry Studies of Wildlife Populations. *Bull. Ecol. Soc. Am.*, 96, 500-507.

Blondel, J., Dervieux, A., Maistre, M. & Perret, P. (1991). Feeding ecology and life history variation of the blue tit in Mediterranean deciduous and sclerophyllous habitats.

Oecologia, 88, 9–14.

Boyer, F., Mercier, C., Bonin, A., Le Bras, Y., Taberlet, P. & Coissac, E. (2016). Obitools: a unix-inspired software package for DNA metabarcoding. *Mol. Ecol. Resour.*, 16, 176-182.

Capilla-Lasheras, P., Dominoni, D.M., Babayan, S.A., O'Shaughnessy, P.J., Mladenova, M., Woodford, L., *et al.* (2017). Elevated immune gene expression is associated with poor reproductive success of urban blue tits. *Front. Ecol. Evol.*, 5, 64.

Clare, E.L., Barber, B.R., Sweeney, B.W., Hebert, P.D.N. & Fenton, M.B. (2011). Eating local: influences of habitat on the diet of litte brown bats (*Myotis lucifugus*). *Mol. Ecol.*, 20, 1772-1780.

Edgar, R.C. (2010). Search and clustering orders of magnitude faster than BLAST. *Bioinformatics*, 26, 2460-2461.

Hijmans, R.J., Williams, E. & Vennes, C. (2012). Geosphere: spherical trigonometry. *R package version*, 1, 2-28.

Jarrett, C., Maillard, F. & Helm, B. (2017). Seasonal trends in the temporal plasticity of breeding in blue tits and great tits in the Loch Lomond area. *Glas. Nat.*, 26.

Joshi, N.A. & Fass, J.N. (2011). Sickle: a sliding- window, adaptive, quality-based trimming tool for FastQ files.

Krüger, F., Clare, E.L., Greif, S., Siemers, B.M., Symondson, W.O.C. & Sommer, R.S. (2014). An integrative approach to detect subtle trophic niche differentiation in the sympatric trawling bat speces *Myotis dasycneme* and *Myotis daubentonii*. *Mol. Ecol.*, 23, 3657-3671.

Machida, R.J., Leray, M., Ho, S-L. & Knowlton, N. (2017). Metazoan mitochondrial gene sequence reference datasets for taxonomic assignment of environmental samples. *Scientific Data*, 4, 170027.

Martin, M. (2011). Cutadapt removes adapter sequences from high-throughput sequencing reads. *EMBnet.journal*, 17, 10-12.

Møller, A. P., Adriaensen, F., Artmyev, A., Bañbura, J., Barba, E. & Biard, C. (2014). Clutch-size variation in Western Palaearctic secondary hole-nesting passerine birds in relation to nest box design. *Methods Ecol. Evol.,* 5, 353-362.

Nikolenko, S.I., Korobeynikov, A.I. & Alekseyev, M.A. (2013). BayesHammer: Bayesian clustering for error correction in single-cell sequencing. *BMC Genomics*, 14(Suppl 1), S7.

Nord, A., Lehmann, M., MacLeod, R., McCafferty, D.J., Nager, R.G., Nilsson, J.-Å., *et al.*

(2016). Evaluation of two methods for minimally invasive peripheral body temperature measurement in birds. *J. Avian Biol.*, 47, 417–427.

Pollock, C.J., Capilla-Lasheras, P., McGill, R., Helm, B. & Dominoni, D.M. (2017). Integrated behavioural and stable isotope data reveal altered diet linked to low breeding success in urban-dwelling blue tits (*Cyanistes caeruleus*). *Sci. Rep.*, 7, 5014.

Razgour, O., Clare, E.L., Zeale, M.R.K., Hanmer, J., Bærholm Schnell, I., Rasmussen, M. *et al.* (2011). High-throughput sequencing offers insight into mechanisms of resource partitioning in cryptic bat species. *Ecol. Evol.*, 1, 556-570.

Schirmer, M., Ijaz, U.Z., D'Amore R, Hall N, Sloan WT, Quince C (2015) Insight into biases and sequencing errors for amplicon sequencing with the Illumina MiSeq platform. Nucleic Acids Research, 43, e37.

Trevelline, B.K., Latta, S.C., Marshall, L.C., Nuttle, T., Porter, B.A. (2016). Molecular analysis of nestling diet in a long-distance Neotropical migrant, the Louisiana Waterthrush (*Parkesia motacilla*). *Auk*, 133, 415-428.

Zepeda-Mendoza, M.L., Bohmann, K., Baez, A.C. and Gilbert, M.T.P., 2016. DAMe: a toolkit for the initial processing of datasets with PCR replicates of double-tagged amplicons for DNA metabarcoding analyses. *BMC research notes*, *9*(1), 255.

Zhang, J., Kobert, K., Flouri, T., Stamatakis, A. (2014). PEAR: a fast and accurate Illumina Paired-End reAd mergeR. *Bioinformatics,* 30(5), 614-620.

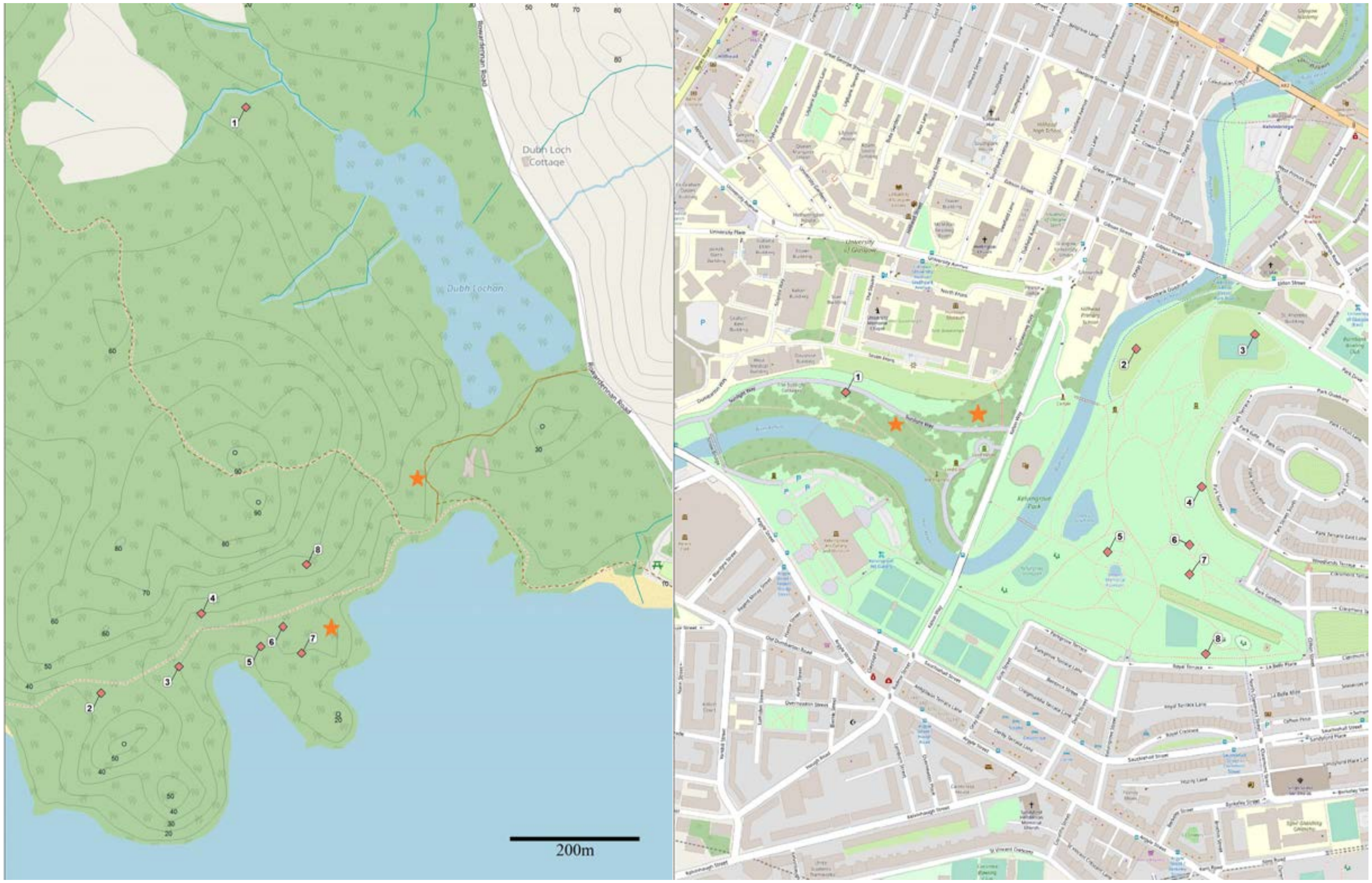

Suppl. Fig. 1. Map of forest site (left) and city site (right). Nestbox locations are indicated by small red squares, and trees used for invertebrate sampling (Jarrett et al., unpubl. data) are indicated with orange stars. Dark green indicates non-coniferous forest, light green is managed lawn.

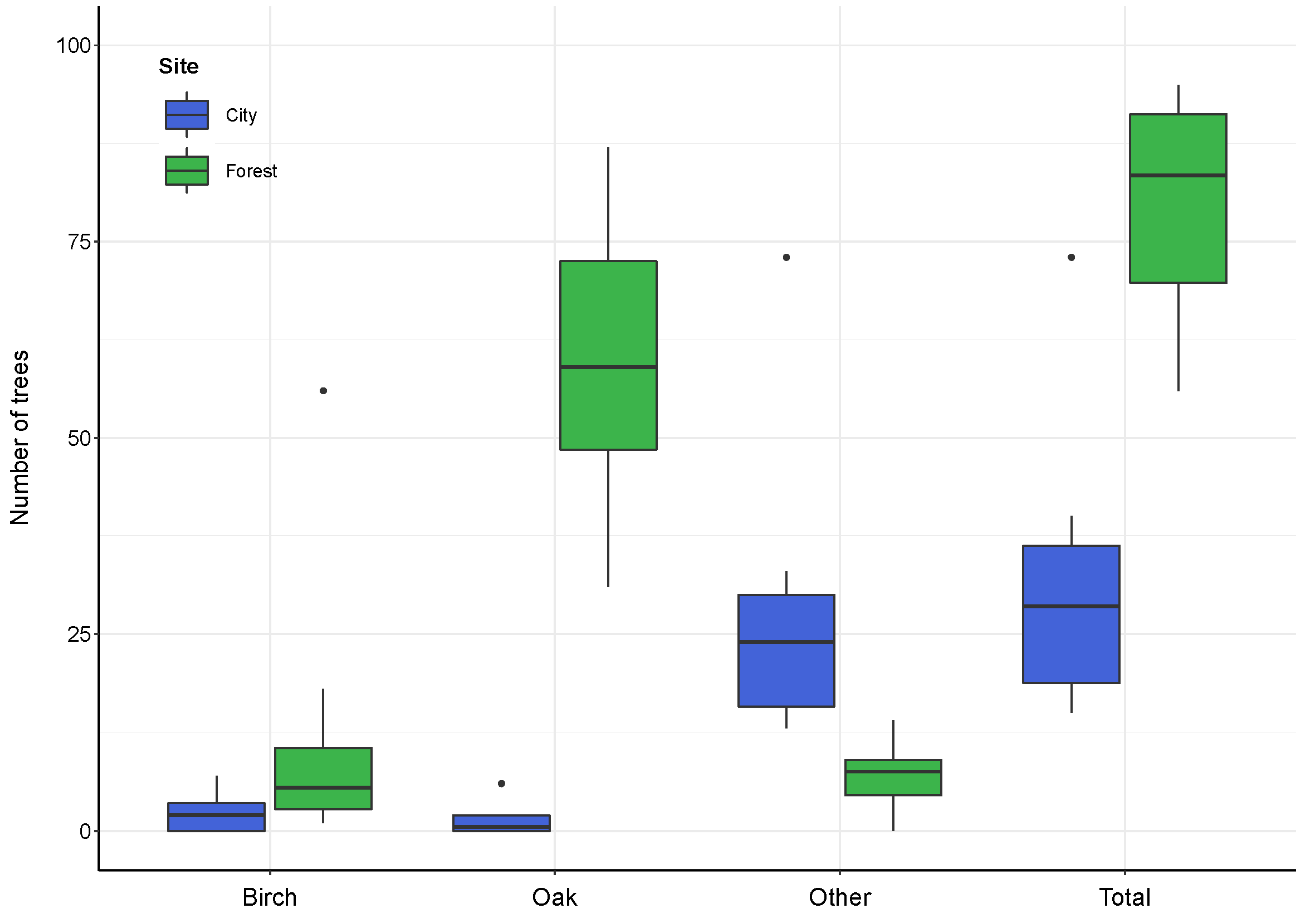

Suppl. Fig. 2. Number of oaks (*Quercus* sp.), birch (*Betula* sp.), other tree species, and total trees in a 35m radius around tracked boxes in the city (blue) and the forest (green). The bold line within each box indicates the median value; the lower and upper limits of the boxes represent the second and third quartiles respectively; and the lines extend to the farthest outliers within 1.5 times the interquartile range.

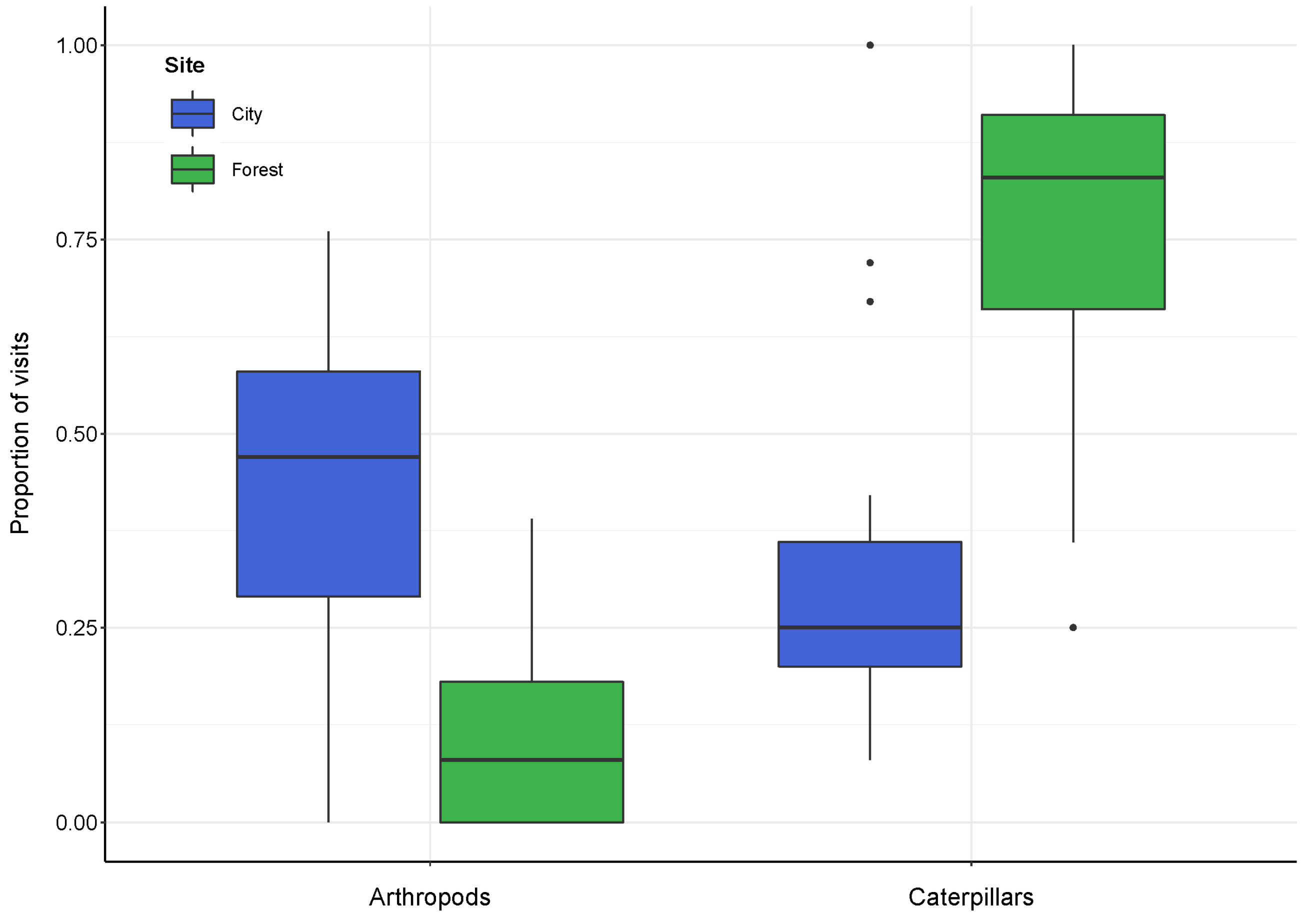

Suppl. Fig. 3. Proportion of visits performed by parents in the city (blue) and the forest (green) in which caterpillars and other arthropods (excluding caterpillars) were delivered to nestlings.

Table S1. Primer sequences used for DNA metabarcoding. The ZBJ-Art primers target the arthropod COI gene, while the rbcL primers target different regions of the plant chloroplast rbcL gene.

**Primer**	**Sequence (5' – 3')***	**Reference**
ZBJ-ArtF1c	**TCGTCGGCAGCGTCAGATGTGTATAAGAGACAG**AGATATTGGAACWTTATATTTTATTTTTGG	Zeale et al. (2011)
ZBJ-ArtR2c	**GTCTCGTGGGCTCGGAGATGTGTATAAGAGACAG**WACTAATCAATTWCCAAATCCTCC	Zeale et al. (2011)
rbcL3	**TCGTCGGCAGCGTCAGATGTGTATAAGAGACAG**CTAAATTGGGATTATCCGCT	This study
rbcL4	**GTCTCGTGGGCTCGGAGATGTGTATAAGAGACAG**AGCGGTCTCTCCAACGCATA	This study
rbcL5	**TCGTCGGCAGCGTCAGATGTGTATAAGAGACAG**TTTCACTCAAGATTGGGTTTCT	This study
rbcL6	**GTCTCGTGGGCTCGGAGATGTGTATAAGAGACAG**ATTTCCCCAAGGGTGTCCTA	This study
rbcL7	**TCGTCGGCAGCGTCAGATGTGTATAAGAGACAG**CTCCTGAATATGAAACCAAAGA	This study
rbcL8	**GTCTCGTGGGCTCGGAGATGTGTATAAGAGACAG**GTAGCAGCGCCCTTTGTAAC	This study

*Bold indicates Illumina adapter overhang

Table S2. Explanatory variables used in full models for analyses, from which we performed model selection by deletion of non-significant variables identified through likelihood ratio tests (LRTs). In the cases where the full model contains only one explanatory variable, we performed LRT tests against the null model. The symbol * between two variables represents an interaction term, and random effects are expressed as (1|Random effect).

Response variable	Type of model	Explanatory variables
Tree density	Negative Binomial GLM	Site
Oak density	Negative Binomial GLM	Site
Birch density	Negative Binomial GLM	Site
Other tree density	Negative Binomial GLM	Site
Proportion of OTUs of Arthropod orders	LMM	Site + date + site*date + (1|nestbox)
Proportion of OTUs of Plant orders	LMM	Site + date + site*date + (1|nestbox)
Provisioning rate per nest and per nestling	LMM	Site + time + date + nestling age + (1|nestbox)
Dietary items provisioned (proportions from footage)	LMM	Site + time + date + nestling age + (1|nestbox)
Volume of delivered caterpillars (footage)	LMM	Site + time + date + nestling age + (1|nestbox)
Foraging distance	LMM	Site + time + sex + number of hatchlings + nestling age + tree density + site* number of hatchlings + (1|nestbox)
Caterpillar biomass delivered	LMM	Site + time + site*foraging distance + (1|nestbox)
Clutch size & number of fledglings	Poisson GLM	Site + date of first egg
Hatching success & fledging success	Binomial GLM	Site + date of first egg
Adult body mass	Gaussian GLM	Site + sex
Nestling body mass (a)	LMM	Site + number of hatchlings + hatch date + (1|nestbox)
Nestling body mass (b)	LMM	Proportion of delivered caterpillars + number of hatchlings + hatch date + (1|nestbox) + (1|site)

Table S3: Summary models of comparisons between an urban and forest site. Shown are minimal adequate models after elimination of non-significant factors, while however retaining site in every model. Table details analyses of a) tree community composition, b) foraging distance, c) video-recorded provisioning, d) faecal metabarcoding, and reproductive outcomes for e) non-tracked boxes and f) tracked boxes. Reference levels are: urban (for site), female for sex, and evening (for time).

a) Tree community composition

Response variable	Explanatory variable	Estimate	Std.Error	t value	p value
Total trees					
	Intercept	3.46	0.12	26.75	<2E-16
	Site	0.9	0.17	5.1	**3.39E-07**
Oak					
	Intercept	0.31	0.3	0.99	0.32
	Site	3.77	0.34	11.09	**<2E-16**
Birch					
	Intercept	0.86	0.44	1.92	5.00E-02
	Site	1.65	0.6	2.74	**6.00E-03**
Other trees					
	Intercept	3.34	0.23	12.41	<2E-16
	Site	-1.41	0.34	-4.06	**4.77E-05**

b) Foraging distance

Response variable	Explanatory variable	Estimate	Std.Error	Df	t value	p value
Foraging distance						
	Intercept	32.1	25.9	15.6	-1.7	0.1
	Site	-17.75	6.69	12.21	-2.65	**0.02**
	Sex	17.88	6.61	11.66	2.7	**0.03**
	Nestling age	2.55	0.86	504.68	2.94	**0.01**
	Number of hatchlings	6.98	2.78	13.91	2.5	**0.05**

c) Video-recorded provisioning

Response variable	Explanatory variable	Estimate	Std.Error	Df	t value	p value
Visits per nest						
	Intercept	29.88	2.18	19.15	13.64	2.56E-11
	Time	-16.5	2.27	33.48	-7.25	**2.32E-08**
	Site	1.16	3.34	9.97	0.35	0.73
Visits per nestling (log)						
	Intercept	1.53	0.07	26.39	21.45	<2E-16
	Time	-0.6	0.09	32.67	-6.77	**1.08E-07**
	Site	-0.06	0.16	14.66	-0.4	0.69
Proportion of caterpillars (log)						
	Intercept	0.27	0.03	11.82	7.95	4.41E-06
	Site	0.28	0.05	12.14	5.95	**6.39E-05**
Proportion of arthropods (log)						
	Intercept	0.33	0.04	9.4	9.44	4.25E-06
	Site	-0.22	0.05	9.64	-4.51	**0.001**

Mean caterpillar volume					
	Intercept	4.11	0.14	11.96	28.44	2.33E-12
	Site	0.59	0.2	11.91	2.94	**0.01**
**Dependence of caterpillar biomass on foraging distance**					
	Intercept	590.4	460.8	8.4	1.3	0.2
	Site	-210.4	800	5.7	-0.26	0.8
	Foraging distance	5.6	11.3	8.3	0.5	0.6
	Site*Foraging distance	54.8	24.5	6.4	2.2	**0.04**
	Time	-0.45	0.1	33.82	-4.26	**3.60E-04**

d) Faecal metabarcoding

Response variable	Explanatory variable	Estimate	Std.Error	t value	p value
Proportion Lepidoptera					
	Intercept	0.93	0.25	3.64	0.002
	Site	0.44	0.05	7.76	**1.93E-06**
	Date	-0.007	0.0004	-1.85	0.08
Proportion Diptera					
	Intercept	-0.2	0.21	-0.95	0.35
	Site	-0.23	0.04	-4.99	**1.95E-04**
	Date	0.007	0.003	2.07	**0.05**
Proportion Coleoptera					
	Intercept	0.13	0.01	10.33	3.24E-08
	Site	-0.09	0.02	-4.66	**3.03E-04**
Proportion Hemiptera					
	Intercept	0.42	0.07	5.59	8.76E-05
	Site*Date	0.008	0.004	2.09	**0.05**
	Date	-0.006	0.001	-4.74	**3.82E-04**
	Site	-0.6	0.27	-2.16	**0.04**
Proportion Hymenoptera					
	Intercept	-0.18	0.04	-4.53	4.67E-04
	Site	-0.03	0.009	-4.23	**8.38E-04**
	Date	0.003	0.0006	5.17	**1.40E-04**
Proportion Plecoptera					
	Intercept	0.16	0.07	2.13	0.04
	Date	-0.002	0.001	-1.93	**0.07**
	Site	-9.78E-05	1.94E-02	-0.005	0.99
Proportion Araneae					
	Intercept	0.04	0.01	3.86	0.001
	Site	-0.008	0.01	-0.47	0.64
Proportion Ephemeroptera					
	Intercept	0.004	0.003	1.29	0.21
	Site	-0.004	0.004	-0.82	0.42
Proportion Sarcoptiformes					
	Intercept	0.007	0.005	1.27	0.22
	Site	-0.005	0.008	-0.59	0.55
Order Fagales					
	Intercept	0.17	0.05	3.04	0.008
	Site	0.3	0.08	3.46	**0.003**
Order Rosales					

	Intercept	0.23	0.05	4.29	6.37E-04
	Site	0.15	0.08	1.82	**0.08**

e) Reproductive Outcomes (non-tracked boxes)

Response variable	Explanatory variable	Estimate	Std.Error	z value	p value
Clutch size (Poisson GLM)					
	Intercept	2.42	0.17	14.61	<2E-16
	Site	0.26	0.07	3.53	**4.09E-04**
	Laying date	-0.01	0.01	-2.57	**0.01**
Hatching success (Binomial GLM)					
	Intercept	-0.23	0.04	-5.30	1.13E-07
	Site	0.12	0.11	1.12	0.26
Fledging success (Binomial GLM)					
	Intercept	-0.25	0.11	-2.15	0.03
	Site	0.15	0.13	1.23	0.22
No. of fledglings (Poisson GLM)					
	Intercept	1.95	0.20	9.55	1.28E-21
	Site	0.54	0.09	5.73	**1.02E-08**
	First egg	-0.02	0.01	-2.69	**0.01**
Nestling body mass at day 13 (LMM)					
	Intercept	11.00	0.28	39.47	2.00E-16
	Site	0.52	0.34	25.70	0.14

f) Reproductive Outcomes (tracked boxes)

Response variable	Explanatory variable	Estimate	Std.Error	z value	p value
Clutch size (Poisson GLM)					
	Intercept	2.12	0.12	17.39	<2E-16
	Site	0.13	0.16	0.83	0.4
Hatching success (Binomial GLM)					
	Intercept	-0.33	0.53	-0.63	0.52
	Site	-0.04	0.29	-0.15	0.88
Fledging success (Binomial GLM)					
	Intercept	-0.2	0.57	-0.36	0.72
	Site	-0.07	0.32	-0.23	0.82
No. of fledglings (Poisson GLM)					
	Intercept	1.85	0.14	13.22	<2E-16
	Site	0.11	0.19	0.57	0.56
Adult body mass (Gaussian GLM)					
	Intercept	11.06	0.13	83.14	2.89E-20
	Site	-0.15	0.27	-0.56	0.58
Nestling body mass at day 13 (LMM)					
	Intercept	9.97	0.22	45.16	1.3E-13
	Site	0.87	0.32	2.67	**0.02**
	Hatch date (quadratic)	-6.33	1.37	-4.63	**7.48E-04**
Nestling body mass at day 13 (LMM)					
	Intercept	9.33	0.52	17.62	0.09
	Proportion of visits with caterpillars*	2.2	0.87	2.52	0.23

Table S4. Taxonomic assignment of all OTUs from nestling faecal samples in the city (blue) and the forest (green). Number of OTU reads refers to the total reads per site (all individuals pooled). OTUs are ordered by decreasing number of reads in the forest.

Phylum	Class	Order	Family	Genus	Species	Number of reads	
Arthropoda	Insecta	Lepidoptera	Geometridae	Hydriomena	*Hydriomena furcata*	13	25091
Arthropoda	Insecta	Lepidoptera	Geometridae	Operophtera	*Operophtera brumata*	3	18389
Streptophyta	Unassigned	Unassigned	Unassigned	Unassigned	Unassigned	19466	9302
Arthropoda	Insecta	Lepidoptera	Geometridae	Operophtera	*Operophtera fagata*	0	6019
Arthropoda	Insecta	Lepidoptera	Geometridae	Erannis	*Erannis defoliaria*	12	4310
Arthropoda	Insecta	Lepidoptera	Noctuidae	Cosmia	*Cosmia trapezina*	8166	4227
Arthropoda	Insecta	Lepidoptera	Geometridae	Agriopis	*Agriopis leucophaearia*	0	4083
Arthropoda	Insecta	Lepidoptera	Noctuidae	Brachylomia	*Brachylomia viminalis*	1	3702
Streptophyta	Unassigned	Rosales	Rosaceae	Unassigned	Unassigned	7382	2461
Arthropoda	Insecta	Lepidoptera	Geometridae	Apocheima	*Apocheima pilosaria*	3031	1401
Arthropoda	Insecta	Lepidoptera	Ypsolophidae	Ypsolopha	*Ypsolopha ustella*	1	1140
Arthropoda	Insecta	Lepidoptera	Tortricidae	Acleris	*Acleris rhombana*	0	920
Arthropoda	Insecta	Lepidoptera	Tortricidae	Tortrix	*Tortrix viridana*	1	902
Arthropoda	Arachnida	Araneae	Anyphaenidae	Anyphaena	*Anyphaena accentuata*	0	763
Arthropoda	Insecta	Lepidoptera	Ypsolophidae	Ypsolopha	*Ypsolopha parenthesella*	0	635
Arthropoda	Insecta	Lepidoptera	Geometridae	Agriopis	*Agriopis marginaria*	0	514
Arthropoda	Insecta	Lepidoptera	Lycaenidae	Quercusia	*Quercusia quercus*	0	411
Arthropoda	Insecta	Lepidoptera	Geometridae	Agriopis	*Agriopis aurantiaria*	0	122
Streptophyta	Unassigned	Fagales	Fagaceae	Unassigned	Unassigned	717	110
Arthropoda	Insecta	Lepidoptera	Geometridae	Erannis	*Erannis defoliaria*	0	100
Arthropoda	Insecta	Lepidoptera	Oecophoridae	Diurnea	*Diurnea lipsiella*	0	92
Arthropoda	Insecta	Coleoptera	Tenebrionidae	Tenebrio	*Tenebrio molitor*	505	90
Arthropoda	Insecta	Lepidoptera	Geometridae	Eupithecia	*Eupithecia abbreviata*	0	83
Arthropoda	Insecta	Diptera	Tachinidae	Lypha	*Lypha dubia*	0	55
Arthropoda	Insecta	Lepidoptera	Geometridae	Operophtera	*Operophtera brumata*	0	55
Arthropoda	Insecta	Coleoptera	Curculionidae	Phyllobius	*Phyllobius oblongus*	40	53
Arthropoda	Insecta	Lepidoptera	Noctuidae	Cosmia	*Cosmia trapezina*	3	48
Arthropoda	Insecta	Lepidoptera	Unassigned	Unassigned	Unassigned	0	45

Phylum	Class	Order	Family	Genus	Species	Number of reads	
Arthropoda	Insecta	Diptera	Rhagionidae	Rhagio	*Rhagio mystaceus*	47	44
Arthropoda	Insecta	Lepidoptera	Geometridae	Crocallis	*Crocallis elinguaria*	0	41
Arthropoda	Insecta	Lepidoptera	Unassigned	Unassigned	Unassigned	0	40
Arthropoda	Insecta	Lepidoptera	Erebidae	Bleptina	Unassigned	4	38
Arthropoda	Insecta	Hemiptera	Miridae	Phylus	*Phylus melanocephalus*	0	37
Arthropoda	Insecta	Lepidoptera	Geometridae	Operophtera	*Operophtera fagata*	0	32
Arthropoda	Insecta	Lepidoptera	Geometridae	Unassigned	Unassigned	28	31
Arthropoda	Insecta	Lepidoptera	Unassigned	Unassigned	Unassigned	0	26
Arthropoda	Insecta	Lepidoptera	Geometridae	Unassigned	Unassigned	0	25
Arthropoda	Insecta	Hymenoptera	Ichneumonidae	Hyposoter	*Hyposoter inquinatus*	0	24
Arthropoda	Insecta	Lepidoptera	Geometridae	Unassigned	Unassigned	0	24
Arthropoda	Insecta	Lepidoptera	Unassigned	Unassigned	Unassigned	0	23
Arthropoda	Insecta	Lepidoptera	Geometridae	Agriopis	*Agriopis leucophaearia*	0	20
Arthropoda	Insecta	Unassigned	Unassigned	Unassigned	Unassigned	0	20
Arthropoda	Insecta	Unassigned	Unassigned	Unassigned	Unassigned	0	20
Arthropoda	Insecta	Lepidoptera	Nolidae	Pseudoips	*Pseudoips prasinanus*	0	19
Arthropoda	Insecta	Lepidoptera	Unassigned	Unassigned	Unassigned	0	19
Arthropoda	Insecta	Lepidoptera	Unassigned	Unassigned	Unassigned	0	19
Arthropoda	Insecta	Lepidoptera	Noctuidae	Unassigned	Unassigned	0	18
Arthropoda	Insecta	Lepidoptera	Unassigned	Unassigned	Unassigned	2	18
Arthropoda	Insecta	Lepidoptera	Geometridae	Euphyia	*Euphyia mesembrina*	0	17
Arthropoda	Insecta	Unassigned	Unassigned	Unassigned	Unassigned	0	17
Arthropoda	Insecta	Lepidoptera	Unassigned	Unassigned	Unassigned	0	17
Arthropoda	Insecta	Lepidoptera	Geometridae	Unassigned	Unassigned	0	17
Arthropoda	Insecta	Lepidoptera	Noctuidae	Lithophane	Unassigned	3	17
Arthropoda	Insecta	Lepidoptera	Unassigned	Unassigned	Unassigned	0	16
Arthropoda	Insecta	Lepidoptera	Geometridae	Unassigned	Unassigned	0	16
Arthropoda	Insecta	Lepidoptera	Unassigned	Unassigned	Unassigned	0	15
Arthropoda	Insecta	Hemiptera	Lachnidae	Cinara	*Cinara pruinosa*	2	14
Arthropoda	Arachnida	Araneae	Clubionidae	Clubiona	*Clubiona canadensis*	0	14
Arthropoda	Arachnida	Araneae	Philodromidae	Philodromus	*Philodromus rufus*	10	14
Arthropoda	Insecta	Lepidoptera	Geometridae	Unassigned	Unassigned	0	14

Phylum	Class	Order	Family	Genus	Species	Number of reads	
Arthropoda	Insecta	Lepidoptera	Geometridae	Unassigned	Unassigned	0	13
Arthropoda	Insecta	Diptera	Phoridae	Unassigned	Unassigned	0	13
Arthropoda	Insecta	Lepidoptera	Unassigned	Unassigned	Unassigned	0	12
Arthropoda	Insecta	Lepidoptera	Geometridae	Unassigned	Unassigned	0	12
Arthropoda	Insecta	Lepidoptera	Geometridae	Unassigned	Unassigned	0	12
Arthropoda	Insecta	Lepidoptera	Geometridae	Unassigned	Unassigned	0	12
Arthropoda	Insecta	Lepidoptera	Tortricidae	Epinotia	*Epinotia trigonella*	0	11
Arthropoda	Insecta	Lepidoptera	Geometridae	Hydriomena	*Hydriomena furcata*	0	11
Arthropoda	Insecta	Unassigned	Unassigned	Unassigned	Unassigned	0	11
Arthropoda	Insecta	Lepidoptera	Unassigned	Unassigned	Unassigned	0	11
Arthropoda	Insecta	Lepidoptera	Unassigned	Unassigned	Unassigned	0	11
Arthropoda	Insecta	Lepidoptera	Geometridae	Unassigned	Unassigned	0	11
Arthropoda	Insecta	Lepidoptera	Geometridae	Unassigned	Unassigned	0	11
Arthropoda	Insecta	Lepidoptera	Ypsolophidae	Unassigned	Unassigned	0	11
Arthropoda	Insecta	Lepidoptera	Elachistidae	Unassigned	Unassigned	0	11
Arthropoda	Insecta	Lepidoptera	Erebidae	Unassigned	Unassigned	0	11
Arthropoda	Insecta	Lepidoptera	Unassigned	Unassigned	Unassigned	0	10
Arthropoda	Insecta	Lepidoptera	Geometridae	Unassigned	Unassigned	0	10
Arthropoda	Insecta	Lepidoptera	Erebidae	Unassigned	Unassigned	0	10
Arthropoda	Insecta	Lepidoptera	Geometridae	Unassigned	Unassigned	0	10
Arthropoda	Insecta	Lepidoptera	Unassigned	Unassigned	Unassigned	18	10
Arthropoda	Insecta	Diptera	Tachinidae	Oswaldia	*Oswaldia muscaria*	0	9
Arthropoda	Insecta	Lepidoptera	Unassigned	Unassigned	Unassigned	0	9
Arthropoda	Insecta	Lepidoptera	Unassigned	Unassigned	Unassigned	0	9
Arthropoda	Insecta	Lepidoptera	Oecophoridae	Unassigned	Unassigned	0	9
Arthropoda	Insecta	Lepidoptera	Geometridae	Unassigned	Unassigned	1	9
Arthropoda	Insecta	Lepidoptera	Gelechiidae	Unassigned	Unassigned	0	9
Arthropoda	Insecta	Lepidoptera	Unassigned	Unassigned	Unassigned	0	9
Arthropoda	Insecta	Lepidoptera	Noctuidae	Orthosia	Unassigned	0	9
Arthropoda	Arachnida	Sarcoptiformes	Parakalummidae	Unassigned	Unassigned	11	9
Arthropoda	Insecta	Lepidoptera	Unassigned	Unassigned	Unassigned	0	8
Arthropoda	Insecta	Lepidoptera	Noctuidae	Unassigned	Unassigned	0	8

Phylum	Class	Order	Family	Genus	Species	Number of reads	
Arthropoda	Insecta	Lepidoptera	Unassigned	Unassigned	Unassigned	0	8
Arthropoda	Arachnida	Araneae	Unassigned	Unassigned	Unassigned	2	8
Arthropoda	Insecta	Unassigned	Unassigned	Unassigned	Unassigned	0	7
Arthropoda	Insecta	Lepidoptera	Unassigned	Unassigned	Unassigned	0	7
Arthropoda	Insecta	Lepidoptera	Unassigned	Unassigned	Unassigned	0	7
Arthropoda	Insecta	Lepidoptera	Noctuidae	Unassigned	Unassigned	0	7
Arthropoda	Insecta	Lepidoptera	Geometridae	Unassigned	Unassigned	1	7
Arthropoda	Insecta	Lepidoptera	Geometridae	Unassigned	Unassigned	0	7
Arthropoda	Insecta	Lepidoptera	Geometridae	Unassigned	Unassigned	0	7
Arthropoda	Insecta	Lepidoptera	Geometridae	Unassigned	Unassigned	0	7
Arthropoda	Insecta	Lepidoptera	Geometridae	Erannis	*Erannis defoliaria*	0	6
Arthropoda	Insecta	Lepidoptera	Geometridae	Erannis	*Erannis defoliaria*	0	6
Arthropoda	Insecta	Lepidoptera	Geometridae	Erannis	*Erannis defoliaria*	0	6
Arthropoda	Insecta	Lepidoptera	Geometridae	Operophtera	*Operophtera brumata*	0	6
Arthropoda	Insecta	Lepidoptera	Unassigned	Unassigned	Unassigned	0	6
Arthropoda	Insecta	Lepidoptera	Unassigned	Unassigned	Unassigned	0	6
Arthropoda	Insecta	Lepidoptera	Unassigned	Unassigned	Unassigned	0	6
Arthropoda	Insecta	Lepidoptera	Scythrididae	Unassigned	Unassigned	0	6
Arthropoda	Insecta	Lepidoptera	Noctuidae	Unassigned	Unassigned	0	6
Arthropoda	Insecta	Lepidoptera	Unassigned	Unassigned	Unassigned	0	6
Arthropoda	Insecta	Lepidoptera	Geometridae	Unassigned	Unassigned	0	6
Arthropoda	Insecta	Lepidoptera	Geometridae	Unassigned	Unassigned	0	6
Arthropoda	Insecta	Lepidoptera	Geometridae	Operophtera	Unassigned	0	6
Arthropoda	Insecta	Lepidoptera	Crambidae	Eulepte	Unassigned	0	6
Arthropoda	Insecta	Lepidoptera	Geometridae	Agriopis	*Agriopis leucophaearia*	0	5
Arthropoda	Insecta	Lepidoptera	Geometridae	Agriopis	*Agriopis leucophaearia*	0	5
Arthropoda	Insecta	Lepidoptera	Tortricidae	Argyroploce	*Argyroploce externa*	0	5
Arthropoda	Insecta	Lepidoptera	Sphingidae	Clanis	*Clanis pratti*	0	5
Arthropoda	Insecta	Diptera	Tachinidae	Cyzenis	*Cyzenis albicans*	0	5
Arthropoda	Insecta	Lepidoptera	Geometridae	Erannis	*Erannis defoliaria*	0	5
Arthropoda	Insecta	Lepidoptera	Geometridae	Operophtera	*Operophtera fagata*	0	5
Arthropoda	Insecta	Lepidoptera	Tortricidae	Ptycholoma	*Ptycholoma lecheana*	163	5

Phylum	Class	Order	Family	Genus	Species	Number of reads	
Arthropoda	Insecta	Unassigned	Unassigned	Unassigned	Unassigned	0	5
Arthropoda	Insecta	Unassigned	Unassigned	Unassigned	Unassigned	0	5
Arthropoda	Insecta	Unassigned	Unassigned	Unassigned	Unassigned	0	5
Arthropoda	Insecta	Lepidoptera	Unassigned	Unassigned	Unassigned	0	5
Arthropoda	Insecta	Lepidoptera	Unassigned	Unassigned	Unassigned	0	5
Arthropoda	Insecta	Lepidoptera	Unassigned	Unassigned	Unassigned	0	5
Arthropoda	Insecta	Lepidoptera	Unassigned	Unassigned	Unassigned	0	5
Arthropoda	Insecta	Hymenoptera	Unassigned	Unassigned	Unassigned	0	5
Arthropoda	Insecta	Lepidoptera	Unassigned	Unassigned	Unassigned	0	5
Arthropoda	Insecta	Lepidoptera	Unassigned	Unassigned	Unassigned	0	5
Arthropoda	Insecta	Lepidoptera	Unassigned	Unassigned	Unassigned	0	5
Arthropoda	Insecta	Lepidoptera	Crambidae	Unassigned	Unassigned	0	5
Arthropoda	Insecta	Lepidoptera	Geometridae	Unassigned	Unassigned	0	5
Arthropoda	Insecta	Lepidoptera	Unassigned	Unassigned	Unassigned	0	5
Arthropoda	Insecta	Lepidoptera	Drepanidae	Unassigned	Unassigned	0	5
Arthropoda	Insecta	Lepidoptera	Unassigned	Unassigned	Unassigned	0	5
Arthropoda	Insecta	Lepidoptera	Noctuidae	Unassigned	Unassigned	1	5
Arthropoda	Insecta	Lepidoptera	Geometridae	Unassigned	Unassigned	0	5
Arthropoda	Insecta	Lepidoptera	Geometridae	Unassigned	Unassigned	0	5
Arthropoda	Insecta	Lepidoptera	Unassigned	Unassigned	Unassigned	0	5
Arthropoda	Insecta	Lepidoptera	Geometridae	Unassigned	Unassigned	0	5
Arthropoda	Arachnida	Araneae	Anyphaenidae	Unassigned	Unassigned	0	5
Arthropoda	Insecta	Lepidoptera	Geometridae	Unassigned	Unassigned	0	5
Arthropoda	Insecta	Lepidoptera	Geometridae	Unassigned	Unassigned	0	5
Arthropoda	Insecta	Lepidoptera	Geometridae	Unassigned	Unassigned	0	5
Arthropoda	Insecta	Lepidoptera	Geometridae	Operophtera	Unassigned	0	5
Arthropoda	Insecta	Lepidoptera	Unassigned	Unassigned	Unassigned	0	5
Arthropoda	Insecta	Hymenoptera	Ichneumonidae	Chorinaeus	Unassigned	0	5
Streptophyta	Unassigned	Unassigned	Unassigned	Unassigned	Unassigned	12	5
Streptophyta	Unassigned	Unassigned	Unassigned	Unassigned	Unassigned	4	5
Arthropoda	Insecta	Unassigned	Unassigned	Unassigned	Unassigned	4	4
Arthropoda	Arachnida	Unassigned	Unassigned	Unassigned	Unassigned	4	4

Phylum	Class	Order	Family	Genus	Species	Number of reads	
Arthropoda	Insecta	Unassigned	Unassigned	Unassigned	Unassigned	3	4
Arthropoda	Insecta	Lepidoptera	Unassigned	Unassigned	Unassigned	1	4
Arthropoda	Insecta	Lepidoptera	Unassigned	Unassigned	Unassigned	4	4
Streptophyta	Unassigned	Rosales	Rosaceae	Unassigned	Unassigned	7	4
Streptophyta	Unassigned	Rosales	Rosaceae	Unassigned	Unassigned	6	4
Arthropoda	Insecta	Lepidoptera	Pyralidae	Galleria	*Galleria mellonella*	6	3
Arthropoda	Insecta	Unassigned	Unassigned	Unassigned	Unassigned	14	3
Arthropoda	Insecta	Diptera	Chironomidae	Unassigned	Unassigned	2	3
Arthropoda	Insecta	Lepidoptera	Noctuidae	Catocala	Unassigned	2	3
Arthropoda	Insecta	Lepidoptera	Geometridae	Cladara	Unassigned	46	3
Arthropoda	Insecta	Plecoptera	Nemouridae	Ostrocerca	Unassigned	5	3
Streptophyta	Unassigned	Rosales	Rosaceae	Unassigned	Unassigned	3	3
Arthropoda	Insecta	Lepidoptera	Noctuidae	Amphipyra	*Amphipyra pyramidoides*	6	2
Arthropoda	Insecta	Lepidoptera	Geometridae	Unassigned	Unassigned	7	2
Arthropoda	Insecta	Lepidoptera	Noctuidae	Unassigned	Unassigned	0	2
Streptophyta	Unassigned	Sapindales	Unassigned	Unassigned	Unassigned	4	2
Streptophyta	Unassigned	Fagales	Fagaceae	Unassigned	Unassigned	4	2
Arthropoda	Insecta	Lepidoptera	Tortricidae	Hedya	*Hedya nubiferana*	5135	1
Arthropoda	Insecta	Lepidoptera	Noctuidae	Orthosia	*Orthosia incerta*	10	1
Arthropoda	Insecta	Unassigned	Unassigned	Unassigned	Unassigned	6	1
Arthropoda	Insecta	Lepidoptera	Unassigned	Unassigned	Unassigned	5	1
Arthropoda	Insecta	Diptera	Unassigned	Unassigned	Unassigned	11	1
Arthropoda	Insecta	Lepidoptera	Unassigned	Unassigned	Unassigned	4	1
Arthropoda	Insecta	Coleoptera	Unassigned	Unassigned	Unassigned	24	1
Streptophyta	Unassigned	Unassigned	Unassigned	Unassigned	Unassigned	7	1
Streptophyta	Unassigned	Fagales	Fagaceae	Unassigned	Unassigned	6	1
Streptophyta	Unassigned	Unassigned	Unassigned	Unassigned	Unassigned	3	1
Streptophyta	Unassigned	Fagales	Fagaceae	Unassigned	Unassigned	3	1
Streptophyta	Unassigned	Fagales	Fagaceae	Unassigned	Unassigned	3	1
Streptophyta	Unassigned	Fagales	Fagaceae	Unassigned	Unassigned	2	1
Streptophyta	Unassigned	Rosales	Rosaceae	Docynia	Unassigned	2	1
Streptophyta	Unassigned	Fagales	Unassigned	Unassigned	Unassigned	1	1

Phylum	Class	Order	Family	Genus	Species	Number of reads	
Streptophyta	Unassigned	Unassigned	Unassigned	Unassigned	Unassigned	1	1
Streptophyta	Unassigned	Fagales	Casuarinaceae	Gymnostoma	Unassigned	1	1
Streptophyta	Unassigned	Fagales	Fagaceae	Unassigned	Unassigned	1	1
Streptophyta	Unassigned	Fabales	Fabaceae	Unassigned	Unassigned	1	1
Unassigned	Unassigned	Unassigned	Unassigned	Unassigned	Unassigned	0	1
Streptophyta	Unassigned	Fagales	Fagaceae	Unassigned	Unassigned	0	1
Streptophyta	Unassigned	Myrtales	Combretaceae	Unassigned	Unassigned	0	1
Streptophyta	Unassigned	Unassigned	Unassigned	Unassigned	Unassigned	0	1
Streptophyta	Unassigned	Unassigned	Unassigned	Unassigned	Unassigned	0	1
Streptophyta	Unassigned	Rosales	Unassigned	Unassigned	Unassigned	0	1
Streptophyta	Unassigned	Unassigned	Unassigned	Unassigned	Unassigned	0	1
Streptophyta	Unassigned	Fagales	Fagaceae	Unassigned	Unassigned	0	1
Streptophyta	Unassigned	Unassigned	Unassigned	Unassigned	Unassigned	0	1
Streptophyta	Unassigned	Fagales	Unassigned	Unassigned	Unassigned	0	1
Streptophyta	Unassigned	Rosales	Rosaceae	Unassigned	Unassigned	0	1
Streptophyta	Unassigned	Rosales	Unassigned	Unassigned	Unassigned	0	1
Streptophyta	Unassigned	Unassigned	Unassigned	Unassigned	Unassigned	0	1
Streptophyta	Unassigned	Fagales	Fagaceae	Unassigned	Unassigned	0	1
Streptophyta	Unassigned	Fagales	Fagaceae	Unassigned	Unassigned	0	1
Streptophyta	Unassigned	Unassigned	Unassigned	Unassigned	Unassigned	0	1
Streptophyta	Unassigned	Rosales	Rosaceae	Unassigned	Unassigned	0	1
Streptophyta	Unassigned	Fagales	Fagaceae	Unassigned	Unassigned	0	1
Streptophyta	Unassigned	Fabales	Fabaceae	Unassigned	Unassigned	0	1
Streptophyta	Unassigned	Fabales	Fabaceae	Unassigned	Unassigned	0	1
Streptophyta	Unassigned	Unassigned	Unassigned	Unassigned	Unassigned	0	1
Arthropoda	Insecta	Coleoptera	Coccinellidae	Calvia	*Calvia quatuordecimguttata*	15	0
Arthropoda	Insecta	Lepidoptera	Geometridae	Campaea	*Campaea margaritaria*	34	0
Arthropoda	Arachnida	Araneae	Clubionidae	Clubiona	*Clubiona comta*	15	0
Arthropoda	Insecta	Lepidoptera	Geometridae	Colotois	*Colotois pennaria*	6	0
Arthropoda	Insecta	Lepidoptera	Tortricidae	Ditula	*Ditula angustiorana*	22	0
Arthropoda	Insecta	Hemiptera	Aphididae	Drepanosiphum	*Drepanosiphum platanoidis*	220	0
Arthropoda	Insecta	Diptera	Syrphidae	Epistrophe	*Epistrophe eligans*	6	0

Phylum	Class	Order	Family	Genus	Species	Number of reads	
Arthropoda	Insecta	Coleoptera	Staphylinidae	Haploglossa	*Haploglossa villosula*	5	0
Arthropoda	Insecta	Lepidoptera	Tortricidae	Hedya	*Hedya nubiferana*	6	0
Arthropoda	Insecta	Ephemeroptera	Ephemeridae	Litobrancha	*Litobrancha recurvata*	45	0
Arthropoda	Insecta	Lepidoptera	Tortricidae	Lorita	*Lorita scarificata*	10	0
Arthropoda	Insecta	Lepidoptera	Tortricidae	Pandemis	*Pandemis cinnamomeana*	20	0
Arthropoda	Insecta	Hemiptera	Aphididae	Periphyllus	*Periphyllus testudinaceus*	5	0
Arthropoda	Insecta	Diptera	Syrphidae	Syrphus	*Syrphus torvus*	12131	0
Arthropoda	Insecta	Unassigned	Unassigned	Unassigned	Unassigned	17	0
Arthropoda	Unassigned	Unassigned	Unassigned	Unassigned	Unassigned	11	0
Arthropoda	Insecta	Unassigned	Unassigned	Unassigned	Unassigned	10	0
Arthropoda	Insecta	Unassigned	Unassigned	Unassigned	Unassigned	126	0
Arthropoda	Insecta	Unassigned	Unassigned	Unassigned	Unassigned	20	0
Arthropoda	Insecta	Unassigned	Unassigned	Unassigned	Unassigned	9	0
Arthropoda	Insecta	Unassigned	Unassigned	Unassigned	Unassigned	790	0
Arthropoda	Insecta	Unassigned	Unassigned	Unassigned	Unassigned	9	0
Arthropoda	Insecta	Diptera	Unassigned	Unassigned	Unassigned	7	0
Arthropoda	Insecta	Diptera	Unassigned	Unassigned	Unassigned	21	0
Arthropoda	Insecta	Diptera	Unassigned	Unassigned	Unassigned	13	0
Arthropoda	Insecta	Lepidoptera	Unassigned	Unassigned	Unassigned	6	0
Arthropoda	Insecta	Lepidoptera	Geometridae	Unassigned	Unassigned	6	0
Arthropoda	Insecta	Diptera	Syrphidae	Unassigned	Unassigned	5	0
Arthropoda	Insecta	Lepidoptera	Tortricidae	Unassigned	Unassigned	9	0
Arthropoda	Insecta	Lepidoptera	Pyralidae	Unassigned	Unassigned	2	0
Arthropoda	Insecta	Lepidoptera	Noctuidae	Unassigned	Unassigned	7	0
Arthropoda	Insecta	Diptera	Syrphidae	Unassigned	Unassigned	5	0
Arthropoda	Insecta	Diptera	Syrphidae	Unassigned	Unassigned	5	0
Arthropoda	Insecta	Diptera	Syrphidae	Unassigned	Unassigned	2259	0
Arthropoda	Insecta	Diptera	Syrphidae	Unassigned	Unassigned	11	0
Arthropoda	Insecta	Diptera	Syrphidae	Syrphus	Unassigned	8290	0
Arthropoda	Arachnida	Araneae	Philodromidae	Philodromus	Unassigned	197	0
Arthropoda	Insecta	Diptera	Unassigned	Unassigned	Unassigned	84	0
Arthropoda	Insecta	Hymenoptera	Ichneumonidae	Unassigned	Unassigned	42	0

Phylum	Class	Order	Family	Genus	Species	Number of reads	
Arthropoda	Insecta	Lepidoptera	Geometridae	Pasiphila	Unassigned	35	0
Arthropoda	Insecta	Lepidoptera	Unassigned	Unassigned	Unassigned	19	0
Arthropoda	Insecta	Hymenoptera	Ichneumonidae	Stictopisthus	Unassigned	10	0
Streptophyta	Unassigned	Picramniales	Picramniaceae	Alvaradoa	*Alvaradoa amorphoides*	1	0
Streptophyta	Unassigned	Fagales	Fagaceae	Castanopsis	*Castanopsis boisii*	1	0
Streptophyta	Unassigned	Santalales	Aptandraceae	Chaunochiton	*Chaunochiton kappleri*	1	0
Proteobacteria	Gammaproteoba	Enterobacterales	Enterobacteriaceae	Enterobacter	*Enterobacter cancerogenus*	1	0
Streptophyta	Unassigned	Malvales	Malvaceae	Goethalsia	*Goethalsia meiantha*	2	0
Streptophyta	Unassigned	Fagales	Fagaceae	Lithocarpus	*Lithocarpus nitidinux*	1	0
Streptophyta	Unassigned	Sapindales	Sapindaceae	Unassigned	Unassigned	19173	0
Streptophyta	Unassigned	Malvales	Malvaceae	Unassigned	Unassigned	586	0
Streptophyta	Unassigned	Asterales	Asteraceae	Unassigned	Unassigned	454	0
Streptophyta	Unassigned	Lamiales	Lamiaceae	Salvia	Unassigned	432	0
Streptophyta	Unassigned	Sapindales	Unassigned	Unassigned	Unassigned	128	0
Streptophyta	Unassigned	Rosales	Unassigned	Unassigned	Unassigned	92	0
Streptophyta	Unassigned	Unassigned	Unassigned	Unassigned	Unassigned	70	0
Streptophyta	Unassigned	Unassigned	Unassigned	Unassigned	Unassigned	65	0
Streptophyta	Unassigned	Fagales	Betulaceae	Unassigned	Unassigned	49	0
Streptophyta	Unassigned	Sapindales	Unassigned	Unassigned	Unassigned	47	0
Streptophyta	Unassigned	Unassigned	Unassigned	Unassigned	Unassigned	38	0
Streptophyta	Unassigned	Fabales	Fabaceae	Trifolium	Unassigned	30	0
Streptophyta	Unassigned	Fabales	Fabaceae	Unassigned	Unassigned	19	0
Streptophyta	Unassigned	Sapindales	Sapindaceae	Unassigned	Unassigned	17	0
Streptophyta	Unassigned	Sapindales	Sapindaceae	Unassigned	Unassigned	14	0
Streptophyta	Unassigned	Malpighiales	Salicaceae	Unassigned	Unassigned	11	0
Streptophyta	Unassigned	Sapindales	Sapindaceae	Unassigned	Unassigned	9	0
Streptophyta	Unassigned	Fagales	Fagaceae	Unassigned	Unassigned	9	0
Streptophyta	Unassigned	Unassigned	Unassigned	Unassigned	Unassigned	6	0
Streptophyta	Unassigned	Unassigned	Unassigned	Unassigned	Unassigned	6	0
Streptophyta	Unassigned	Fagales	Fagaceae	Unassigned	Unassigned	6	0
Streptophyta	Unassigned	Sapindales	Sapindaceae	Unassigned	Unassigned	5	0
Streptophyta	Unassigned	Rosales	Unassigned	Unassigned	Unassigned	4	0

Phylum	Class	Order	Family	Genus	Species	Number of reads	
Streptophyta	Unassigned	Sapindales	Unassigned	Unassigned	Unassigned	4	0
Streptophyta	Unassigned	Sapindales	Unassigned	Unassigned	Unassigned	4	0
Streptophyta	Unassigned	Fagales	Fagaceae	Unassigned	Unassigned	4	0
Streptophyta	Unassigned	Sapindales	Sapindaceae	Acer	Unassigned	4	0
Streptophyta	Unassigned	Sapindales	Sapindaceae	Unassigned	Unassigned	4	0
Streptophyta	Unassigned	Fagales	Fagaceae	Unassigned	Unassigned	4	0
Streptophyta	Unassigned	Sapindales	Sapindaceae	Unassigned	Unassigned	3	0
Streptophyta	Unassigned	Unassigned	Unassigned	Unassigned	Unassigned	3	0
Streptophyta	Unassigned	Rosales	Rosaceae	Unassigned	Unassigned	3	0
Streptophyta	Unassigned	Sapindales	Unassigned	Unassigned	Unassigned	3	0
Streptophyta	Unassigned	Unassigned	Unassigned	Unassigned	Unassigned	3	0
Streptophyta	Unassigned	Sapindales	Sapindaceae	Unassigned	Unassigned	3	0
Streptophyta	Unassigned	Fagales	Fagaceae	Unassigned	Unassigned	3	0
Streptophyta	Unassigned	Fagales	Fagaceae	Unassigned	Unassigned	3	0
Streptophyta	Unassigned	Fagales	Fagaceae	Unassigned	Unassigned	3	0
Streptophyta	Unassigned	Apiales	Apiaceae	Hansenia	Unassigned	2	0
Streptophyta	Unassigned	Fagales	Fagaceae	Unassigned	Unassigned	2	0
Streptophyta	Unassigned	Rosales	Rosaceae	Unassigned	Unassigned	2	0
Streptophyta	Unassigned	Sapindales	Sapindaceae	Unassigned	Unassigned	2	0
Streptophyta	Unassigned	Unassigned	Unassigned	Unassigned	Unassigned	2	0
Streptophyta	Unassigned	Unassigned	Unassigned	Unassigned	Unassigned	2	0
Streptophyta	Unassigned	Unassigned	Unassigned	Unassigned	Unassigned	2	0
Streptophyta	Unassigned	Sapindales	Sapindaceae	Unassigned	Unassigned	2	0
Streptophyta	Unassigned	Unassigned	Unassigned	Unassigned	Unassigned	2	0
Streptophyta	Unassigned	Sapindales	Sapindaceae	Unassigned	Unassigned	2	0
Streptophyta	Unassigned	Sapindales	Sapindaceae	Unassigned	Unassigned	2	0
Streptophyta	Unassigned	Unassigned	Unassigned	Unassigned	Unassigned	2	0
Streptophyta	Unassigned	Unassigned	Unassigned	Unassigned	Unassigned	2	0
Streptophyta	Unassigned	Fagales	Fagaceae	Unassigned	Unassigned	2	0
Streptophyta	Unassigned	Unassigned	Unassigned	Unassigned	Unassigned	2	0
Streptophyta	Unassigned	Fagales	Fagaceae	Unassigned	Unassigned	2	0
Streptophyta	Unassigned	Fagales	Fagaceae	Unassigned	Unassigned	2	0

Phylum	Class	Order	Family	Genus	Species	Number of reads	
Streptophyta	Unassigned	Sapindales	Sapindaceae	Unassigned	Unassigned	2	0
Proteobacteria	Unassigned	Unassigned	Unassigned	Unassigned	Unassigned	2	0
Streptophyta	Unassigned	Apiales	Apiaceae	Unassigned	Unassigned	1	0
Streptophyta	Unassigned	Boraginales	Cordiaceae	Unassigned	Unassigned	1	0
Streptophyta	Unassigned	Unassigned	Unassigned	Unassigned	Unassigned	1	0
Streptophyta	Unassigned	Boraginales	Cordiaceae	Unassigned	Unassigned	1	0
Streptophyta	Unassigned	Asterales	Asteraceae	Unassigned	Unassigned	1	0
Streptophyta	Unassigned	Asterales	Asteraceae	Unassigned	Unassigned	1	0
Streptophyta	Unassigned	Lamiales	Unassigned	Unassigned	Unassigned	1	0
Streptophyta	Unassigned	Sapindales	Sapindaceae	Unassigned	Unassigned	1	0
Streptophyta	Unassigned	Unassigned	Unassigned	Unassigned	Unassigned	1	0
Streptophyta	Unassigned	Unassigned	Unassigned	Unassigned	Unassigned	1	0
Streptophyta	Liliopsida	Poales	Poaceae	Unassigned	Unassigned	1	0
Streptophyta	Unassigned	Unassigned	Unassigned	Unassigned	Unassigned	1	0
Streptophyta	Unassigned	Unassigned	Unassigned	Unassigned	Unassigned	1	0
Streptophyta	Unassigned	Unassigned	Unassigned	Unassigned	Unassigned	1	0
Streptophyta	Unassigned	Malpighiales	Trigoniaceae	Unassigned	Unassigned	1	0
Streptophyta	Unassigned	Unassigned	Unassigned	Unassigned	Unassigned	1	0
Streptophyta	Unassigned	Sapindales	Sapindaceae	Unassigned	Unassigned	1	0
Streptophyta	Unassigned	Unassigned	Unassigned	Unassigned	Unassigned	1	0
Streptophyta	Unassigned	Sapindales	Sapindaceae	Unassigned	Unassigned	1	0
Streptophyta	Unassigned	Unassigned	Unassigned	Unassigned	Unassigned	1	0
Streptophyta	Unassigned	Sapindales	Sapindaceae	Unassigned	Unassigned	1	0
Streptophyta	Unassigned	Unassigned	Unassigned	Unassigned	Unassigned	1	0
Streptophyta	Unassigned	Sapindales	Sapindaceae	Unassigned	Unassigned	1	0
Streptophyta	Unassigned	Unassigned	Unassigned	Unassigned	Unassigned	1	0
Streptophyta	Unassigned	Sapindales	Sapindaceae	Unassigned	Unassigned	1	0
Streptophyta	Unassigned	Sapindales	Unassigned	Unassigned	Unassigned	1	0
Streptophyta	Unassigned	Unassigned	Unassigned	Unassigned	Unassigned	1	0
Streptophyta	Unassigned	Unassigned	Unassigned	Unassigned	Unassigned	1	0
Streptophyta	Unassigned	Sapindales	Sapindaceae	Unassigned	Unassigned	1	0
Streptophyta	Unassigned	Sapindales	Sapindaceae	Unassigned	Unassigned	1	0

Phylum	Class	Order	Family	Genus	Species	Number of reads	
Streptophyta	Unassigned	Sapindales	Anacardiaceae	Unassigned	Unassigned	1	0
Streptophyta	Unassigned	Sapindales	Sapindaceae	Unassigned	Unassigned	1	0
Streptophyta	Unassigned	Sapindales	Sapindaceae	Unassigned	Unassigned	1	0
Streptophyta	Unassigned	Ranunculales	Menispermaceae	Unassigned	Unassigned	1	0
Streptophyta	Unassigned	Unassigned	Unassigned	Unassigned	Unassigned	1	0
Streptophyta	Unassigned	Unassigned	Unassigned	Unassigned	Unassigned	1	0
Streptophyta	Unassigned	Unassigned	Unassigned	Unassigned	Unassigned	1	0
Streptophyta	Unassigned	Rosales	Rosaceae	Unassigned	Unassigned	1	0
Streptophyta	Unassigned	Unassigned	Unassigned	Unassigned	Unassigned	1	0
Streptophyta	Unassigned	Unassigned	Unassigned	Unassigned	Unassigned	1	0
Streptophyta	Unassigned	Sapindales	Sapindaceae	Unassigned	Unassigned	1	0
Streptophyta	Unassigned	Unassigned	Unassigned	Unassigned	Unassigned	1	0
Streptophyta	Unassigned	Sapindales	Sapindaceae	Unassigned	Unassigned	1	0
Streptophyta	Unassigned	Unassigned	Unassigned	Unassigned	Unassigned	1	0
Streptophyta	Unassigned	Unassigned	Unassigned	Unassigned	Unassigned	1	0
Streptophyta	Unassigned	Unassigned	Unassigned	Unassigned	Unassigned	1	0
Streptophyta	Unassigned	Unassigned	Unassigned	Unassigned	Unassigned	1	0
Streptophyta	Unassigned	Sapindales	Sapindaceae	Unassigned	Unassigned	1	0
Streptophyta	Unassigned	Unassigned	Unassigned	Unassigned	Unassigned	1	0
Streptophyta	Unassigned	Sapindales	Sapindaceae	Unassigned	Unassigned	1	0
Streptophyta	Unassigned	Sapindales	Sapindaceae	Unassigned	Unassigned	1	0
Streptophyta	Unassigned	Sapindales	Sapindaceae	Unassigned	Unassigned	1	0
Streptophyta	Unassigned	Sapindales	Sapindaceae	Unassigned	Unassigned	1	0
Streptophyta	Unassigned	Fabales	Polygalaceae	Unassigned	Unassigned	1	0
Streptophyta	Liliopsida	Alismatales	Potamogetonaceae	Unassigned	Unassigned	1	0
Streptophyta	Unassigned	Sapindales	Unassigned	Unassigned	Unassigned	1	0
Streptophyta	Unassigned	Sapindales	Anacardiaceae	Unassigned	Unassigned	1	0
Streptophyta	Unassigned	Sapindales	Sapindaceae	Unassigned	Unassigned	1	0
Streptophyta	Unassigned	Sapindales	Sapindaceae	Unassigned	Unassigned	1	0
Streptophyta	Unassigned	Sapindales	Sapindaceae	Unassigned	Unassigned	1	0
Streptophyta	Unassigned	Sapindales	Sapindaceae	Unassigned	Unassigned	1	0
Streptophyta	Unassigned	Unassigned	Unassigned	Unassigned	Unassigned	1	0

Phylum	Class	Order	Family	Genus	Species	Number of reads	
Streptophyta	Unassigned	Fagales	Fagaceae	Unassigned	Unassigned	1	0
Streptophyta	Unassigned	Unassigned	Unassigned	Unassigned	Unassigned	1	0
Streptophyta	Unassigned	Unassigned	Unassigned	Unassigned	Unassigned	1	0
Streptophyta	Unassigned	Malvales	Malvaceae	Unassigned	Unassigned	1	0
Streptophyta	Unassigned	Sapindales	Unassigned	Unassigned	Unassigned	1	0
Streptophyta	Unassigned	Sapindales	Sapindaceae	Unassigned	Unassigned	1	0
Streptophyta	Unassigned	Unassigned	Unassigned	Unassigned	Unassigned	1	0
Streptophyta	Liliopsida	Unassigned	Unassigned	Unassigned	Unassigned	1	0
Streptophyta	Unassigned	Unassigned	Unassigned	Unassigned	Unassigned	1	0
Streptophyta	Unassigned	Fagales	Fagaceae	Unassigned	Unassigned	1	0
Streptophyta	Unassigned	Unassigned	Unassigned	Unassigned	Unassigned	1	0
Streptophyta	Unassigned	Unassigned	Unassigned	Unassigned	Unassigned	1	0
Streptophyta	Unassigned	Unassigned	Unassigned	Unassigned	Unassigned	1	0
Streptophyta	Unassigned	Unassigned	Unassigned	Unassigned	Unassigned	1	0
Streptophyta	Unassigned	Unassigned	Unassigned	Unassigned	Unassigned	1	0
Streptophyta	Unassigned	Rosales	Rosaceae	Unassigned	Unassigned	1	0
Streptophyta	Unassigned	Unassigned	Unassigned	Unassigned	Unassigned	1	0
Streptophyta	Unassigned	Unassigned	Unassigned	Unassigned	Unassigned	1	0
Streptophyta	Unassigned	Unassigned	Unassigned	Unassigned	Unassigned	1	0
Streptophyta	Unassigned	Unassigned	Unassigned	Unassigned	Unassigned	1	0
Streptophyta	Unassigned	Fagales	Fagaceae	Unassigned	Unassigned	1	0
Streptophyta	Unassigned	Fagales	Fagaceae	Unassigned	Unassigned	1	0
Streptophyta	Unassigned	Unassigned	Unassigned	Unassigned	Unassigned	1	0
Streptophyta	Unassigned	Unassigned	Unassigned	Unassigned	Unassigned	1	0
Streptophyta	Unassigned	Fabales	Fabaceae	Unassigned	Unassigned	1	0
Streptophyta	Unassigned	Unassigned	Unassigned	Unassigned	Unassigned	1	0
Streptophyta	Unassigned	Unassigned	Unassigned	Unassigned	Unassigned	1	0
Streptophyta	Unassigned	Unassigned	Unassigned	Unassigned	Unassigned	1	0
Streptophyta	Unassigned	Unassigned	Unassigned	Unassigned	Unassigned	1	0
Streptophyta	Unassigned	Fagales	Fagaceae	Unassigned	Unassigned	1	0
Streptophyta	Unassigned	Rosales	Rosaceae	Unassigned	Unassigned	1	0
Streptophyta	Unassigned	Unassigned	Unassigned	Unassigned	Unassigned	1	0

Phylum	Class	Order	Family	Genus	Species	Number of reads	
Streptophyta	Unassigned	Unassigned	Unassigned	Unassigned	Unassigned	1	0
Streptophyta	Unassigned	Fagales	Fagaceae	Unassigned	Unassigned	1	0
Streptophyta	Unassigned	Unassigned	Unassigned	Unassigned	Unassigned	1	0
Streptophyta	Unassigned	Fagales	Fagaceae	Unassigned	Unassigned	1	0
Streptophyta	Unassigned	Rosales	Rosaceae	Unassigned	Unassigned	1	0
Streptophyta	Unassigned	Malpighiales	Euphorbiaceae	Unassigned	Unassigned	1	0
Streptophyta	Unassigned	Rosales	Rosaceae	Unassigned	Unassigned	1	0
Streptophyta	Unassigned	Unassigned	Unassigned	Unassigned	Unassigned	1	0
Streptophyta	Unassigned	Unassigned	Unassigned	Unassigned	Unassigned	1	0
Streptophyta	Unassigned	Unassigned	Unassigned	Unassigned	Unassigned	1	0
Streptophyta	Unassigned	Malpighiales	Unassigned	Unassigned	Unassigned	1	0
Streptophyta	Unassigned	Unassigned	Unassigned	Unassigned	Unassigned	1	0
Streptophyta	Unassigned	Rosales	Rosaceae	Unassigned	Unassigned	1	0
Streptophyta	Unassigned	Unassigned	Unassigned	Unassigned	Unassigned	1	0
Streptophyta	Unassigned	Unassigned	Unassigned	Unassigned	Unassigned	1	0
Streptophyta	Unassigned	Unassigned	Unassigned	Unassigned	Unassigned	1	0
Streptophyta	Unassigned	Unassigned	Unassigned	Unassigned	Unassigned	1	0
Streptophyta	Unassigned	Unassigned	Unassigned	Unassigned	Unassigned	1	0
Streptophyta	Unassigned	Fagales	Fagaceae	Unassigned	Unassigned	1	0
Streptophyta	Unassigned	Unassigned	Unassigned	Unassigned	Unassigned	1	0
Streptophyta	Unassigned	Fagales	Fagaceae	Unassigned	Unassigned	1	0
Streptophyta	Unassigned	Unassigned	Unassigned	Unassigned	Unassigned	1	0
Streptophyta	Unassigned	Fagales	Fagaceae	Unassigned	Unassigned	1	0
Streptophyta	Unassigned	Unassigned	Unassigned	Unassigned	Unassigned	1	0
Streptophyta	Unassigned	Unassigned	Unassigned	Unassigned	Unassigned	1	0
Streptophyta	Unassigned	Unassigned	Unassigned	Unassigned	Unassigned	1	0
Streptophyta	Unassigned	Unassigned	Unassigned	Unassigned	Unassigned	1	0
Streptophyta	Unassigned	Unassigned	Unassigned	Unassigned	Unassigned	1	0
Streptophyta	Unassigned	Unassigned	Unassigned	Unassigned	Unassigned	1	0
Streptophyta	Unassigned	Fagales	Fagaceae	Unassigned	Unassigned	1	0
Streptophyta	Unassigned	Fagales	Fagaceae	Unassigned	Unassigned	1	0
Streptophyta	Unassigned	Unassigned	Unassigned	Unassigned	Unassigned	1	0

Phylum	Class	Order	Family	Genus	Species	Number of reads	
Streptophyta	Unassigned	Fagales	Fagaceae	Unassigned	Unassigned	1	0
Streptophyta	Unassigned	Fagales	Fagaceae	Unassigned	Unassigned	1	0
Streptophyta	Unassigned	Unassigned	Unassigned	Unassigned	Unassigned	1	0
Streptophyta	Unassigned	Unassigned	Unassigned	Unassigned	Unassigned	1	0
Streptophyta	Unassigned	Fagales	Fagaceae	Unassigned	Unassigned	1	0
Streptophyta	Unassigned	Fagales	Fagaceae	Unassigned	Unassigned	1	0
Streptophyta	Unassigned	Unassigned	Unassigned	Unassigned	Unassigned	1	0
Streptophyta	Unassigned	Fabales	Fabaceae	Unassigned	Unassigned	1	0
Streptophyta	Unassigned	Unassigned	Unassigned	Unassigned	Unassigned	1	0
Streptophyta	Unassigned	Fagales	Fagaceae	Unassigned	Unassigned	1	0
Streptophyta	Unassigned	Rosales	Rosaceae	Unassigned	Unassigned	1	0
Streptophyta	Unassigned	Unassigned	Unassigned	Unassigned	Unassigned	1	0
Streptophyta	Unassigned	Sapindales	Sapindaceae	Unassigned	Unassigned	1	0
Streptophyta	Unassigned	Fagales	Fagaceae	Unassigned	Unassigned	1	0
Streptophyta	Unassigned	Unassigned	Unassigned	Unassigned	Unassigned	1	0
Streptophyta	Unassigned	Unassigned	Unassigned	Unassigned	Unassigned	1	0
Streptophyta	Unassigned	Rosales	Unassigned	Unassigned	Unassigned	1	0
Streptophyta	Unassigned	Unassigned	Unassigned	Unassigned	Unassigned	1	0
Streptophyta	Unassigned	Unassigned	Unassigned	Unassigned	Unassigned	1	0
Streptophyta	Unassigned	Rosales	Urticaceae	Boehmeria	Unassigned	1	0
Streptophyta	Unassigned	Fagales	Fagaceae	Unassigned	Unassigned	1	0
Streptophyta	Unassigned	Unassigned	Unassigned	Unassigned	Unassigned	1	0
Streptophyta	Unassigned	Sapindales	Unassigned	Unassigned	Unassigned	1	0
Streptophyta	Unassigned	Sapindales	Sapindaceae	Unassigned	Unassigned	1	0
Streptophyta	Unassigned	Fagales	Fagaceae	Unassigned	Unassigned	1	0
Streptophyta	Unassigned	Unassigned	Unassigned	Unassigned	Unassigned	1	0
Streptophyta	Unassigned	Sapindales	Unassigned	Unassigned	Unassigned	1	0
Streptophyta	Unassigned	Fagales	Fagaceae	Unassigned	Unassigned	1	0
Streptophyta	Unassigned	Fagales	Fagaceae	Unassigned	Unassigned	1	0
Streptophyta	Unassigned	Unassigned	Unassigned	Unassigned	Unassigned	1	0
Streptophyta	Unassigned	Sapindales	Sapindaceae	Unassigned	Unassigned	1	0
Streptophyta	Unassigned	Unassigned	Unassigned	Unassigned	Unassigned	1	0

Phylum	Class	Order	Family	Genus	Species	Number of reads	
Streptophyta	Unassigned	Sapindales	Sapindaceae	Unassigned	Unassigned	1	0
Streptophyta	Unassigned	Unassigned	Unassigned	Unassigned	Unassigned	1	0
Streptophyta	Unassigned	Unassigned	Unassigned	Unassigned	Unassigned	1	0
Streptophyta	Unassigned	Fagales	Fagaceae	Unassigned	Unassigned	1	0
Streptophyta	Unassigned	Fagales	Fagaceae	Unassigned	Unassigned	1	0
Streptophyta	Unassigned	Fagales	Unassigned	Unassigned	Unassigned	1	0
Streptophyta	Unassigned	Fabales	Fabaceae	Pterocarpus	*Pterocarpus indicus*	1	0
Streptophyta	Unassigned	Sapindales	Anacardiaceae	Rhus	*Rhus chinensis*	4	0
Streptophyta	Unassigned	Rosales	Rosaceae	Rubus	*Rubus idaeus*	1	0
Streptophyta	Unassigned	Sapindales	Sapindaceae	Ungnadia	*Ungnadia speciosa*	1	0
